# Axonal tau reduction ameliorates tau and amyloid pathology in a mouse model of Alzheimer’s disease

**DOI:** 10.1186/s40035-025-00499-0

**Published:** 2025-07-29

**Authors:** Abdolhossein Zare, Saeede Salehi, Jakob M. Bader, Anna-Lena Wiessler, Manuela Prokesch, Vincent Albrecht, Carmen Villmann, Matthias Mann, Michael Briese, Michael Sendtner

**Affiliations:** 1 Institute of Clinical Neurobiology, University Hospital Wuerzburg, Würzburg, Germany; 2 Department of Proteomics and Signal Transduction, Max Planck Institute of Biochemistry, Martinsried, Munich, Germany; 3 Scantox Neuro GmbH, Grambach, Austria; 4 NNF Center for Protein Research, Faculty of Health Sciences, University of Copenhagen, Copenhagen, Denmark

**Keywords:** Alzheimer’s disease, Tau, *Mapt*, Amyloid, hnRNP R, Antisense oligonucleotide

## Abstract

**Background:**

Pathological deposition of hyperphosphorylated tau in the brain closely correlates with the course of Alzheimer’s disease (AD). Tau pathology occurs in axons of affected neurons and tau removal from axons might thus be an early intervention strategy.

**Methods:**

We investigated the role of the RNA-binding protein hnRNP R in axonal localization and local translation of *Mapt* mRNA in neurons cultured from hnRNP R knockout mice. hnRNP R knockout mice were crossed with 5×FAD mice, an AD mouse model, and the effects of hnRNP R loss on the deposition of phospho-tau and amyloid-β plaques were evaluated. We designed antisense oligonucleotides (MAPT-ASOs) to block the binding of hnRNP R to *Mapt* mRNA. Cultured mouse and human neurons were treated with MAPT-ASOs and axonal *Mapt* mRNA and tau protein levels were quantified. MAPT-ASO was injected intracerebroventricularly into 5×FAD mice followed by quantification of phospho-tau aggregates and amyloid-β plaques in their brains. Protein changes in brains of 5×FAD mice treated with the MAPT-ASO were measured by mass spectrometry.

**Results:**

*Mapt* mRNA and tau protein were reduced in axons but not cell bodies of primary neurons cultured from hnRNP R knockout mice. Brains of 5×FAD mice deficient for hnRNP R contained less phospho-tau aggregates and amyloid-β plaques in the cortex and hippocampus. Treatment of neurons with MAPT-ASOs to block hnRNP R binding to *Mapt* similarly reduced axonal tau levels. Intracerebroventricular injection of a MAPT-ASO reduced the phospho-tau and plaque load and prevented neurodegeneration in the brains of 5×FAD mice, accompanied by rescue of proteome alterations.

**Conclusion:**

Lowering of tau selectively in axons thus represents an innovative therapeutic perspective for treatment of AD and other tauopathies.

**Supplementary Information:**

The online version contains supplementary material available at 10.1186/s40035-025-00499-0.

## Introduction

Alzheimer’s disease (AD) is a progressive neurodegenerative disorder primarily leading to memory loss and also affecting other cognitive functions over time. Pathologically, AD is characterized by the extracellular deposition of amyloid-β (Aβ) protein as senile plaques and the intracellular accumulation of neurofibrillary tangles containing hyperphosphorylated tau protein. While Aβ plaques are present widespread in brains of AD patients, the temporal and spatial formation of tangles correlates more closely with cognitive impairments and the progression of the disease [1–6]. Conversely, a low tangle burden is associated with healthy brain aging [7]. Tau depletion prevents Aβ-induced neurotoxicity [8–10] and alleviates memory defects of AD mouse models [11, 12], indicating a causal role of tau accumulation in AD pathogenesis. Thus, preventing tau pathology has become a main research avenue for AD therapy development [13].

Current tau-targeting therapeutic strategies use antibodies, vaccines or antisense oligonucleotides (ASOs) to reduce total tau levels [13]. Beyond that, tau aggregation inhibitors and approaches to prevent tau hyperphosphorylation such as phosphatase activators and kinase inhibitors are being tested [14]. Additionally, tau is modified by the addition of O-linked β-N-acetylglucosamine (O-GlcNAc) at serine/threonine residues, which thereby protects these sites from phosphorylation [15]. Preventing O-GlcNAc removal by inhibiting O-GlcNAcase (OGA) has thus been pursued as a therapeutic strategy and is currently being tested in clinical trials [16]. However, targeting enzymes that modulate many proteins might give rise to unwanted side effects, potentially limiting the success of these strategies. ASOs targeting the tau-encoding *MAPT* mRNA for RNase H-mediated degradation have proven effective in lowering tau protein levels in cultured neurons and mouse brain following intracerebroventricular administration [17, 18]. ASO-mediated tau reduction in the PS19 tauopathy mouse model expressing mutant P301S human tau prolonged survival and alleviated behavioural deficits [17]. However, globally reducing tau in the brain for prolonged times, particularly during aging, might have adverse consequences for neuronal functions. For instance, while tau knockout mice appear phenotypically normal at middle age due to compensatory upregulation of other microtubule-associated proteins, they show synaptic defects and memory impairments at old ages [19]. Additionally, acute tau knockdown in the hippocampus of 7-month-old mice causes motor and memory impairments accompanied by synaptic abnormalities [20]. Furthermore, treatment of PS19 mice with an antibody targeting total tau worsened their motor impairment [21]. Thus, more targeted approaches are needed to remove tau from subcellular sites at which tau pathology originates.

In AD, oligomeric and hyperphosphorylated tau accumulates in axons and at pre- and postsynaptic terminals [22, 23]. In presynaptic compartments, pathological tau associates with synaptic vesicles via Synaptogyrin-3, impairing neurotransmitter release [24, 25]. Postsynaptically, hyperphosphorylated tau aberrantly accumulates in dendritic spines and affects glutamate receptor trafficking [26]. In the hippocampus of AD patients, axonal tau pathology precedes tau aggregation in somatodendritic regions, suggesting that formation of axonal tau deposits is an early event in the pathological AD cascade [27]. Beyond that, tau is secreted upon neuronal activation through interaction with synaptic vesicle proteins [28, 29], facilitating spreading of tau pathology axonally and transsynaptically between brain regions in AD [30–33]. In neurons, *Mapt* mRNA is transported into axons and locally translated into tau protein [34–36]. Thus, selectively reducing axonal tau by blocking axonal *Mapt* transport might represent an effective strategy for ameliorating AD pathology.

Mechanisms contributing to axonal *Mapt* mRNA localization and local tau synthesis, and their contribution to AD pathology are not well understood. The objective of our study was to determine the role of the neuronal RNA-binding protein heterogeneous nuclear ribonucleoprotein R (hnRNP R) in axonal localization and local translation of *Mapt* mRNA. To do so, we cultured primary neurons from hnRNP R knockout mice and assessed axonal levels of *Mapt* mRNA and tau. We then crossed hnRNP R knockout mice with 5×FAD mice to evaluate the effects of hnRNP R depletion on their plaque and phospho-tau burden in the brain. We designed MAPT-ASOs for blocking the association between hnRNP R and *Mapt* and measured axonal *Mapt* and tau levels in mouse and human neurons treated with these MAPT-ASOs. Finally, 5×FAD mice were injected intracerebroventricularly with a MAPT-ASO to assess its effects on tau and amyloid pathology. Together, our results demonstrate the effectiveness of ASO-mediated axonal tau reduction as a new therapeutic strategy for AD treatment.

## Methods

### Animals

Mice were housed in the animal facility of the Institute of Clinical Neurobiology at the University Hospital Wuerzburg. Mice were maintained on a 12/12 h day/night cycle under controlled conditions at 20–22 °C and 55%–65% humidity with food and water in abundant supply. The 5×FAD mouse strain used for this research project, B6.Cg-Tg(APPSwFlLon,PSEN1*M146L*L286V)6799Vas/Mmjax, RRID:MMRRC_034848-JAX, was obtained from the Mutant Mouse Resource and Research Center (MMRRC) at the Jackson Laboratory, an NIH-funded strain repository. The 5×FAD mouse strain was donated to the MMRRC by Robert Vassar, Ph.D., Northwestern University. The 5×FAD mice overexpress human APP with the familial AD mutations K670N/M671L, I716V and V717I and human presenilin-1 with the familial AD mutations M146L and L286V. *Hnrnpr*^−/−^ mice were generated by CRISPR/Cas9-mediated removal of a genomic region encompassing *Hnrnpr* exons 1–5 [37].

### Primary embryonic mouse motoneurons

Isolation and enrichment of primary mouse motoneurons were performed as previously described [38]. Briefly, lumbar spinal cords were isolated from E13 mouse embryos, and motoneurons were enriched by p75NTR antibody (clone MLR2) panning. Motoneurons were plated on coverslips or in culture dishes coated with poly-DL-ornithine hydrobromide (PORN; P8638, Sigma-Aldrich, Burlington, MA) and laminin-111 (23017–015, Thermo Fisher Scientific, Waltham, MA). Motoneurons were maintained at 37 °C, 5% CO_2_ in Neurobasal medium (Gibco, Thermo Fisher Scientific) supplemented with 2% B27 (Gibco, Thermo Fisher Scientific), 2% heat-inactivated horse serum (Thermo Fisher Scientific), 1% GlutaMAX (Gibco, Thermo Fisher Scientific) and 5 ng/mL of brain-derived neurotrophic factor (BDNF). Medium was replaced one day after plating and then every other day. For compartmentalization, motoneurons were grown in microfluidic chambers (SND 150, Xona Microfluidics, Triangle Park, NC) using a BDNF gradient (20 ng/mL BDNF in axonal compartments) as described previously [39].

### Hippocampal and cortical neuron culture

Cortex and hippocampi were dissected from E18 and P1 mice and immersed in dissection medium (Hanks’ Balanced Salt Solution (Gibco, Thermo Fisher Scientific) containing 1 mmol/L sodium pyruvate (Gibco, Thermo Fisher Scientific), 0.1% glucose and 10 mmol/L HEPES pH 7.3) containing 0.1% trypsin (Worthington Biochemical Corp., Lakewood, NJ) for 20 min at 37 °C. Hippocampi were washed three times with dissection medium followed by addition of DNase (0.1%; AppliChem, Darmstadt, Germany) and incubation for 5 min at room temperature. After two washes with dissection medium, trypsin inhibitor (0.1%; Sigma-Aldrich) was added and hippocampi were triturated. Cells were centrifuged at 300 × *g* for 3 min, washed in dissection medium followed by centrifugation and resuspension in Neurobasal medium containing 2% B27, 1% penicillin/streptomycin, 1% GlutaMAX and 1% heat-inactivated horse serum. Cells were plated on poly-*L*-lysine (P2636, Sigma-Aldrich)- and laminin-111-coated glass coverslips for 1 h followed by incubation in Neurobasal medium containing 2% B27, 1% penicillin/streptomycin and 1% GlutaMAX for 6–25 days in vitro (DIV). Half of the medium was replaced every four days. For compartmentalization, hippocampal neurons were grown in microfluidic chambers (IND 150, Xona Microfluidics) and axon growth was induced by adding BDNF (20 ng/mL) to the axonal side.

### Generation of induced pluripotent stem cell (iPSC)-derived motoneurons

For the generation of iPSC-derived motoneurons, we used the previously reported human iPSC-line 34D6 (control iPSC-line #1) [40], which was kindly provided by Dr. Selvaraj and Prof. Chandran (University of Edinburgh, UK). Motoneurons were differentiated according to a previously published protocol with some modifications [41]. Briefly, iPSCs were expanded in mTeSR Plus medium (05825, STEMCELL Technologies, Vancouver, BC, Canada) on Matrigel (356234, Corning Inc., Corning, NY)-coated dishes. Cells were passaged at 70% confluency using ReLeSR reagent (05872, STEMCELL Technologies). After passaging, cells were cultured in the presence of ROCK inhibitor (10 µmol/L; 130–106–538, Miltenyi Biotec, Bergisch Gladbach, Germany) for 24 h. For initiation of neuronal induction, iPSCs were maintained in mTeSR Plus medium supplemented with 10 µmol/L SB431542 (13031, Cayman Chemical Co., Ann Arbor, MI), 1 µmol/L dorsomorphin homolog 1 (4126, R&D Systems, Minneapolis, MN), 3 µmol/L CHIR99021 (13122, Cayman Chemical Company) and 0.5 µmol/L purmorphamine (PMA; 10009634, Cayman Chemical Company). On day 2, medium was replaced by N2B27 medium containing Neurobasal medium, Dulbecco’s modified Eagle’s medium F-12 (DMEM/F-12; Gibco, Thermo Fisher Scientific), 1% B27, 0.5% N-2 Supplement (17502048, Gibco, Thermo Fisher Scientific), and 1% penicillin/streptomycin/glutamine (Gibco, Thermo Fisher Scientific) supplemented with 10 µmol/L SB431542, 1 µmol/L dorsomorphin homolog 1, 3 µmol/L CHIR99021 and 0.5 µmol/L PMA. On day 4, medium was replaced by expansion medium consisting of N2B27 medium supplemented with 3 µmol/L CHIR99021, 0.5 µmol/L PMA and 150 µmol/L ascorbic acid (A4544, Sigma-Aldrich). Confluent cells were passaged and maintained in suspension on uncoated dishes. Embryoid bodies were picked and triturated with a 1 mL pipette into smaller pieces on day 6. Subsequently, the cells were plated in expansion medium on Matrigel-coated dishes. The resulting neural precursor cells (NPCs) were passaged once every week with Accutase (A1110501, Thermo Fisher Scientific) and expanded for at least 15 passages in expansion medium. Medium was replaced every other day. To induce motoneuron patterning, NPCs were kept for 9 days in N2B27 medium supplemented with 1 µmol/L PMA. On day 2 of patterning, 1 µmol/L retinoic acid (72,264, STEMCELL Technologies) was added to the medium. The medium was replaced every other day. For differentiation, the medium was replaced with N2B27 medium supplemented with 10 ng/mL glia-derived neurotrophic factor (G-240, Alomone Labs), 10 ng/mL BDNF (167450–02–STD, Tebu-bio) and 500 µmol/L dibutyryl-cAMP (73886, STEMCELL Technologies) and motoneurons were maintained for 25 DIV.

### ASOs

ASOs were synthesized with full phosphorothioate backbone and 2’-*O*-methyl groups at all nucleotides (Metabion, Planegg, Germany). For ASO treatment, cells were incubated with 10 µmol/L ASO or Scramble control oligonucleotide for 15 min at 37 °C prior to plating. Mouse hippocampal, cortical and motoneurons were treated with ASO on DIV 0. Human neurons were dissociated on DIV 4 during differentiation followed by ASO treatment.

### RNA immunoprecipitation

For RNA immunoprecipitation, neurons were cultured on laminin-111-coated 6-cm dishes or in microfluidic chambers for 7 DIV. Neurons were washed once with Dulbecco’s Phosphate Buffered Saline (DPBS, without MgCl_2_ and CaCl_2_; D8537, Sigma-Aldrich) and lysed in lysis buffer (10 mmol/L HEPES pH 7.0, 100 mmol/L KCl, 5 mmol/L MgCl_2_, 0.5% NP-40). Lysates were incubated on ice for 20 min and, for motoneurons, centrifuged at 20,000 × *g* for 10 min at 4 °C. Magnetic Dynabeads Protein A (Thermo Fisher Scientific) was bound to 1 μg hnRNP R antibody or 1 μg rabbit IgG control (Table S1) in 300 µL wash buffer (20 mmol/L Tris–HCl, 150 mmol/L KCl, 2 mmol/L MgCl_2_, 0.1% NP-40) for 1 h at room temperature by rotation. The antibody-bound beads were washed twice with wash buffer, and 300 μL lysate was added to the beads and incubated for 2 h at 4 °C by rotation. Beads were washed twice with 500 µl wash buffer. For motoneurons, total RNA was extracted from the input sample and beads by adding 300 μL buffer A1 (NucleoSpin RNA kit, Macherey–Nagel) and 300 μL absolute ethanol followed by RNA extraction according to the manufacturer’s instructions. For hippocampal neurons cultured in microfluidic chambers, total RNA from somatodendritic and axonal compartments was purified by adding 300 μL extraction buffer (PicoPure RNA Isolation Kit, Thermo Fisher Scientific) and 300 μL absolute ethanol to input and beads followed by RNA extraction according to the manufacturer’s instructions. RNA was reverse-transcribed with random hexamers using the First Strand cDNA Synthesis Kit (Thermo Fisher Scientific). Reverse transcription reactions were diluted 1:5 in water and transcript levels were measured by qPCR. Relative RNA binding was calculated using the ΔΔCt method with normalization to input levels. Oligonucleotides are listed in Table S1.

### Fluorescence in situ hybridization (FISH)

*Mapt* FISH was performed with the ViewRNA ISH Cell Assay kit (QVC0001, Invitrogen) as follows. Motoneurons were grown for 5–6 DIV on glass coverslips (0111500, Marienfeld GmbH, Lauda-Königshofen, Germany) coated with PORN and laminin-111. For hippocampal neurons, cells were grown for 6 DIV on glass coverslips coated with poly-*L*-lysine and laminin-111. Cells were washed three times with RNase-free DPBS and fixed for 10 min in paraformaldehyde lysine phosphate (PLP) buffer (pH 7.4) containing 4% paraformaldehyde (PFA) (28908, Thermo Fisher Scientific), 5.4% glucose and 0.01 mol/L sodium metaperiodate. After three DPBS washes, cells were permeabilized with the detergent solution provided with the kit for 4 min. Cells were then incubated with the *Mapt* probe (VB1-13897-VC, Thermo Fisher Scientific) diluted 1:100 in the supplied buffer overnight at 40 °C. The following day, pre-amplifier, amplifier, and label probe solutions diluted 1:25 in the respective buffers were added to the cells sequentially for 1 h each at 40 °C. Three washing steps were performed with the supplied wash buffer before each incubation and after the last one. Next, cells were washed twice with DPBS followed by 4′,6-diamidino-2-phenylindole (DAPI) staining and immunofluorescence staining of tubulin.

Locked nucleic acid (LNA)-oligonucleotide-based FISH detection of MAPT-ASO2 in the brains of injected 5×FAD mice was performed as follows. Brain sections were washed three times with PBS and post-fixed in 4% PFA for 15 min. PFA was removed and samples were incubated with 100% cold methanol for 10–15 min and 70% ethanol for 10 min. Ethanol was aspirated and samples were incubated with 1 mol/L Tris–HCl pH 8.0 (AM9855G, Thermo Fisher Scientific) for 5 min. Tris–HCl was aspirated and samples were incubated with the LNA-modified oligonucleotide diluted to a final concentration of 2 ng/μL in hybridization buffer containing 2 × SSC (AM9770, Thermo Fisher Scientific), 0.1 mg/mL yeast tRNA (R5636, Sigma‐Aldrich), 0.005% Ultrapure™ BSA (50 mg/mL) (AM2618, Thermo Fisher Scientific), 10% dextran sulphate (D8906, Sigma‐Aldrich) and 25% formamide (F7503, Sigma‐Aldrich) at 37 °C for 5 h. The following oligonucleotide was used for detection of MAPT-ASO2: 5’-Cy3-A(+ T)TT(+ C)AT(+ C)TT(+ T)CC(+ A)AA(+ T)TG(+ A)-3’ (where ( +) denotes LNA). After hybridization, samples were washed once with 4 × SSC and once with 2 × SSC. Brain sections were incubated with DAPI (Sigma‐Aldrich) diluted 1:1000 in DPBS for 10 min.

### Puromycin labeling-proximity ligation assay (Puro-PLA)

PLA was carried out using the Duolink InSitu Orange Starter Kit Mouse/Rabbit (DUO92102, Sigma-Aldrich) as described before [42]. Briefly, motoneurons grown for 6 DIV on laminin-111-coated glass coverslips were treated with 10 µg/mL puromycin (P8833, Sigma-Aldrich) for 10 min at 37 °C. As negative control experiments, puromycin was omitted or cells were pre-treated with 100 µg/mL cycloheximide (C4859, Sigma-Aldrich) for 30 min before puromycin treatment. Cells were washed twice with DPBS, fixed in PLP buffer for 10 min followed by permeabilization and washing. Cells were blocked for 1 h at 37 °C and incubated overnight at 4 °C with anti-puromycin and anti-tau antibodies (Table S1) diluted in blocking buffer. PLA probes were applied at 1:5 dilution for 1 h at 37 °C followed by ligation for 30 min at 37 °C and amplification for 100 min at 37 °C. Cells were fixed again for 10 min at room temperature in PLP, washed with DPBS, and processed further for immunofluorescence and DAPI staining.

### Immunofluorescence staining

Motoneurons were cultured on laminin-111- and PORN-coated glass coverslips for 11 DIV. For hippocampal neurons, cells were cultured on laminin-111- and poly-*L*-lysine-coated glass coverslips for 22–25 DIV. Cells were washed twice with DPBS and fixed with 4% PFA at room temperature for 15 min followed by permeabilization with 0.1% Triton X-100 at room temperature for 20 min. After three washes in DPBS, cells were blocked in a blocking buffer containing 4% donkey serum at room temperature for 1 h. Primary antibodies diluted in blocking solution were applied onto coverslips and incubated at 4 °C overnight followed by incubation with secondary antibodies at room temperature for 1 h and counterstaining with DAPI. Coverslips were washed and mounted using FluorSave Reagent (345789, Merck). Antibodies are listed in Table S1.

### Intracerebroventricular ASO injections

ASO injections were performed by Scantox Neuro GmbH (Grambach, Austria). 5×FAD mice aged 8 months received either MAPT-ASO2 (1.8 µg/day) or vehicle (artificial cerebrospinal fluid, aCSF). ASO or vehicle was injected intracerebroventricularly using Alzet minipumps. For the continuous infusion via Alzet minipums, pump model 2006 (volume 200 µL, flowrate 0.15 µL/h) was used allowing an infusion for 42 days. After implantation of Alzet minipumps, health monitoring was performed three times per day for three days and once daily for additional 11 days. For Alzet pump implantation, the fur of the anesthetized animal was shaved on the head and the placement area of the pump. The skin of both areas was disinfected. Eyes were covered with an eye ointment for protection. The animal was placed in a stereotaxic apparatus, on a heating pad to prevent hypothermia. The depth of anesthesia was verified by the toe pinch and eye blink reflex. A midline incision of the scalp was made and the skull was carefully cleaned to make skull suture lines visible. Thereafter, a hole was drilled above the target region, using Bregma as a reference point. The following coordinates were used: AP, -0.5; ML, 1.0; DV, 2.2 mm. An approximately 1-cm-long incision was made on the side chosen for the pump placement. Hemostatic forceps were inserted into the incision underneath the skin to generate a pocket for the pump. The pocket was 1 cm longer than the pump itself. The pump, which was attached to the catheter and the brain cannula, was inserted into the subcutaneous pocket with the delivery port from the pump heading towards the cannula site. For fixation of the cannula, cyanoacrylate adhesive was used. After implantation of the cannula and the pump, wounds were closed with a surgical suture and if necessary, tissue adhesive was applied on the suture. In addition to standard analgesia (Buprenorphine), Carprofen was administered subcutaneously before surgery, as well as after surgery for 2 days. Postoperative care was provided by weighing of animals after surgery, subcutaneous injection of 0.9% saline during wake-up time, provision of wet food for 1 week after surgery and provision of water bottles with long drinking caps and food on the ground during the whole study.

Eight weeks after Alzet pump implantation, animals were terminally anesthetized by injection of pentobarbital (600 mg/kg, dosing 10 μL/g body weight). Animals were transcardially perfused with 0.9% saline. Following perfusion, brains were removed and hemisected on a cooled surface. The right hemibrain was fixed by immersion in freshly prepared 4% PFA in phosphate buffer (pH 7.4) for 2 h at room temperature. Fixed hemibrains were then transferred to 15% sucrose/PBS until samples were sunk to the bottom of the tube (usually overnight) at 4 °C to ensure cryoprotection. Tissue blocks were then trimmed as needed, transferred to cryomolds, embedded in OCT medium and frozen in dry ice-cooled isopentane. Tissue samples were cut into 30-µm sagittal sections using a cryostat (CM1950, Leica, Wetzlar, Germany) and then processed further for immunohistochemical and FISH analysis.

### Immunohistochemistry

Mice were deeply anesthetized and transcardially perfused with 0.1 mol/L phosphate buffer (PB) (pH 7.4) and 4% PFA in PB. Brains were dissected and post-fixed overnight in PFA at 4 °C. Samples were washed, embedded in 6% agarose cubes, and cut into 50-µm coronal sections using a vibratome (VT1000S, Leica). For antibody labeling, sections were washed in PBS, blocked and permeabilized with 4% donkey serum and 0.3% Triton X-100 in PBS for 2 h at room temperature, and subsequently incubated with the primary antibodies at 4 °C for 48 h in blocking solution. Brain sections were allocated randomly for staining with different primary antibodies. After three washes with PBS containing 0.3% Triton X-100, sections were incubated with secondary antibodies for 2 h at room temperature and washed again three times with PBS containing 0.3% Triton X-100. For Thioflavin S (ThS) staining, sections were incubated in 500 µmol/L Thioflavin S (T1892, Sigma-Aldrich) dissolved in 50% ethanol for 7 min. Then, sections were DAPI-stained, mounted onto object slides and embedded with FluorSave Reagent. Antibodies are listed in Table S1.

### Calcium imaging

To analyze spontaneous activity, DIV 21 hippocampal neurons were incubated with 5 µmol/L Oregon Green-bound calcium indicator BAPTA 1-AM (OGB1, Thermo Fisher Scientific) for 15 min at 37 °C. During measurements, cells were supplied with HEPES-buffered aCSF (4.5 mmol/L KCl, 2.5 mmol/L NaH_2_PO_4_, 1 mmol/L MgCl_2_, 2 mmol/L CaCl_2_, 120 mmol/L NaCl, 10 mmol/L HEPES, 25 mmol/L glucose, pH 7.4 adjusted with NaOH) using a peristaltic pump at 37 °C. A BX51WI upright microscope (Olympus) equipped with a 20 × water-immersion objective UMPLanFL NA 0.5 and a pE-4000 fluorescence illumination system (CoolLED, Andover, UK) was used for imaging at 10 Hz with a Rolera XR Mono fast 1394 CCD (Qimaging, Surrey, Canada) camera. For each condition, three individual cultures and three videos per culture were measured. Videos were captured in 3000 frames (300 s) with the software Streampix 4.0 (NorPix, Montreal, QC, Canada) at a rate of 10 frames per second and a binning of 2.

To define regions of interest (ROIs), the Fiji plugin “StarDist” was used with a threshold of 0.65 to identify neurons using star-convex shapes [43]. The NeuralActivityCubic NA^3^ [44] was used to analyze the fluorescent intensity in a.u. calculating calcium activity peaks/events. Parameters used for analysis were “signal-to-noise” of 2.5, “noise-window-size” of 200, “signal-average-threshold” of 10, “include variance” turned to 30, and “minimum activity counts” of 2. With the number of counted total activities, the spontaneous calcium activity per minute per neuron was calculated.

### Protein fractionation

Dissected cortex and hippocampus tissues were lysed and homogenized in 1 mL lysis buffer 1 (10 mmol/L Tris–HCl pH 7.4, 150 mmol/L NaCl, 1% Triton X-100) per 100 mg tissue. Tissue lysates were incubated on ice for 15 min followed by sonication with 15 pulses at 40% amplitude on an ultrasonic processor (UP50H, Hielscher, Teltow, Germany). Samples were centrifuged at 20,000 × *g* for 10 min at 4 °C and the supernatant was collected as the soluble fraction. The pellet was washed with lysis buffer 1 and solubilized in 500 µL lysis buffer 2 (50 mmol/L Tris–HCl pH 8.5, 8 mol/L urea, 2% SDS) per 100 mg tissue on a thermomixer for 30 min at 37 °C and 300 rpm. Pellet samples were then sonicated with 15 pulses at 40% amplitude. Samples were mixed with Laemmli buffer, boiled and subjected to SDS-PAGE, followed by immunoblot analysis. Antibodies are listed in Table S1.

### Sample preparation for proteomics

Soluble cortex and hippocampus fractions were precipitated with acetone and resuspended in 200 µL of lysis buffer containing 10% (*v*/*v*) acetonitrile, 10 mmol/L Tris(2-carboxyethyl)phosphine, 40 mmol/L chloroacetamide, and 50 mmol/L Tris/HCl pH 8.0. The pellets were solubilized by two cycles of boiling at 92 °C for 10 min, sonication in a Bioruptor device (Diagenode) for 10 intervals of 30 s at high intensity, and pipetting through a small 1-mm orifice. To cortical samples, 50 µg of trypsin and LysC were added. To hippocampal samples with a smaller size of the protein pellet, only half of these amounts were added. Proteins were digested at 37 °C overnight. The digestion solution was acidified by addition of 100 µL of 5% trifluoroacetic acid (TFA) and 0.1% formic acid for peptide cleanup. For that purpose, four 10-gauge discs of a styrene divinylbenzene-reversed phase sulfonate (SDB-RPS) resin (Empore) in the STAGE-tip format were used [45]. In brief, peptides were loaded by centrifugation at 1000 × *g* for 3 min and washed with 200 µL of 1% TFA in isopropanol and 0.2% TFA in water, and then spun to dryness. Peptides were eluted in 60 µL of 1% ammonia in 50% acetonitrile and the eluate was evaporated in a vacuum concentrator at 60 °C for 2 h. The peptides were acidified by addition of 30 µL 5% TFA 0.1% formic acid and the concentration was determined by a combination of UV spectrometry (Nanodrop 2000, Thermo Fisher Scientific) and comparison of preliminary mass spectrometric data to those of a reference tryptic digest of HeLa cells. For final measurements, 200 ng of sample peptides were loaded onto Evotips according to the manufacturer’s instructions [46]. In brief, Evotips were soaked in 1-propanol, washed with 0.1% formic acid in acetonitrile, once more soaked with 1-propanol, and washed in 0.1% formic acid in water, loaded with peptides, and again washed in 0.1% formic acid in water.

### LC–MS/MS data acquisition

Mass spectrometric data were acquired by the Orbitrap Astral (Thermo Fisher Scientific) coupled to an Evosep One chromatography device. Peptides were separated on an 8 cm C18 column (Aurora Rapid, IonOpticks, Collingwood, VIC, Australia) at 50 °C over the 21 min gradient built into the Evosep One system. At the interface of chromatography and mass spectrometer, peptides were ionized by an electrospray. A FAIMS device (High-Field Asymmetric Waveform Ion Mobility Spectrometry, Thermo Fisher Scientific) with a compensation voltage of − 40 V was used to filter ions entering the mass spectrometer. The mass spectrometer was operated in data-independent acquisition mode covering a precursor mass range of 380–980. Survey spectra were acquired in the Orbitrap analyzer at a resolution of 240,000 and fragment spectra with the Astral analyzer. The scan range for fragment spectra was set to 150–2000 Th. The precursor windows were adjusted to the complexity of the proteome. Tissue samples were measured with 300 windows of 2 Th allowing a maximum ion injection time of 3 ms per window. Plasma samples were measured with 200 windows of 3 Th allowing a longer ion injection time of 7 ms per window.

### Mass spectrometric raw data processing

To quantify protein abundances across samples, we used the DIA-NN software suite version 1.8.1 in library-free mode [47]. Tissue and plasma samples were processed separately. Spectra were searched against the UniProtKB mouse FASTA database including Swiss-Prot and TrEMBL entries as well as isoforms downloaded in June 2024. Default DIA-NN parameters were utilized unless stated otherwise. Cysteine carbamidomethylation, N-terminal methionine excision and acetylation, as well as oxidation of methionine were enabled. The mass accuracy for MS1 and MS2 and the scan window parameter were set to the mean of those values recommended in the log file of an initial DIA-NN search which had the ‘unrelated runs’ option enabled. The quantities matrices option was enabled and the protein group matrix was used for further analysis.

### Bioinformatic analysis of proteomic data

The protein abundances were log2-transformed and analysed in the Perseus analysis suite version 1.6.15.0 [48]. Hippocampal and cortical proteomes were separately filtered for proteins with observations in at least three out of five samples in at least one of the biological conditions (ASO- or aCSF-injected). Remaining missing values were imputed from a normal distribution around the detection limit modelled on the present protein intensities, using the default Perseus and the total matrix mode. Differential expression was investigated with a Student’s* t*-test between ASO-injected and aCSF-injected samples in log2 space, followed by a permutation-based false discovery rate control using an s0 paramater of 0.1. Proteins above the cutoff line in the Volcano plots have a *P*_adj_ (q-value) below 5%. Proteins that were significantly regulated using this threshold in both hippocampus and cortex were filtered and the fold differences across the two brain regions were correlated in a scatter plot.

### Image acquisition and data analysis

Images were acquired on an Olympus Fluoview 1000 confocal system equipped with the following objectives: 10 × (NA: 0.25), 20 × (NA: 0.75), 40 × (oil differential interference contrast, NA: 1.30), or 60 × (oil differential interference contrast, NA: 1.35). Images were obtained with the Olympus FV10-ASW imaging software for visualization using 405, 473, 559, and 633 nm lasers. The resulting images (Olympus.oib format) were processed using ImageJ software as part of the Fiji package [49]. For FISH, maximum intensity projections were created from 0.3 μm z-stacks. For intensity measurements, raw images were projected using ImageJ and mean grey values were measured after background subtraction. For motoneurons, axonal tau intensity was measured in 20 µm-long proximal and distal regions and, for hippocampal neurons, in 20 µm-long distal regions. For axon length measurements, neurons were immunostained with anti-tubulin antibodies and imaged on a Keyence BZ-8000 K fluorescence microscope equipped with a standard colour camera using a 20 × 0.7-NA objective. The length of the longest axon branch was quantified using ImageJ. Axon collaterals were not considered for the analysis. Brain sections were imaged on a fluorescence microscope (Axio Imager 2, Zeiss) and z-stack images were adjusted for brightness and contrast using ImageJ software. 6E10, anti-Iba1 and AT8 immunosignals were quantified semiautomatically using the ImageJ threshold and particle analysis plugin after background subtraction. Hippocampal area was measured in ImageJ. For quantification of DAPI^+^ and NeuN^+^ cells, a rectangle of the same size was placed over a region of CA1 in ImageJ and the number of DAPI^+^ cells that were positive for NeuN was counted.

### Nest building

Nesting activity was scored as previously described [50]. Mice were supplied with six cellulose pads (5 × 4 cm; Lohmann & Rauscher GmbH & Co. KG) placed throughout the cages 1.5 h before the onset of the night cycle. Nests were scored the next morning as follows: 1—pads are still distributed throughout the cage; 2—pads are centred but intact; 3—pads are centred and partially torn; 4—identifiable nest, more than 75% of the pads are torn; 5—clear nest, pads are more than 90% torn.

### Quantitative PCR

For qPCR analysis of compartmentalized motoneurons, total RNA was extracted using the PicoPure RNA Isolation Kit (KIT0204, Thermo Fisher Scientific) and reverse-transcribed using the First Strand cDNA Synthesis Kit (K1612, Thermo Fisher Scientific). qPCR reactions were set up with the Luminaris HiGreen qPCR Master Mix (K0994, Thermo Fisher Scientific) and run on a LightCycler 96 (Roche, Basel, Switzerland). qPCR primers (Table S1) were designed using the online Primer3Plus software. An annealing temperature of 60 °C was used for all primers. Two technical qPCR replicates were set up for each sample and their Ct values were averaged before normalization and statistical analyses. Relative expression was calculated using the ΔΔCt method.

### Quantification and statistical analysis

All statistical analyses were performed using GraphPad Prism version 9 for Windows (GraphPad Software, San Diego, CA). Data are presented as violin plots (median and quartiles marked) or bar graphs when *n* < 10. No data were excluded from the analyses. Data adherence to normal distribution was tested prior to statistical analysis. Individual test names are included in the figure legends. Venn diagrams were generated using BioVenn [51].

## Results

### Depletion of hnRNP R reduces axonal tau levels

**Fig. 1:**
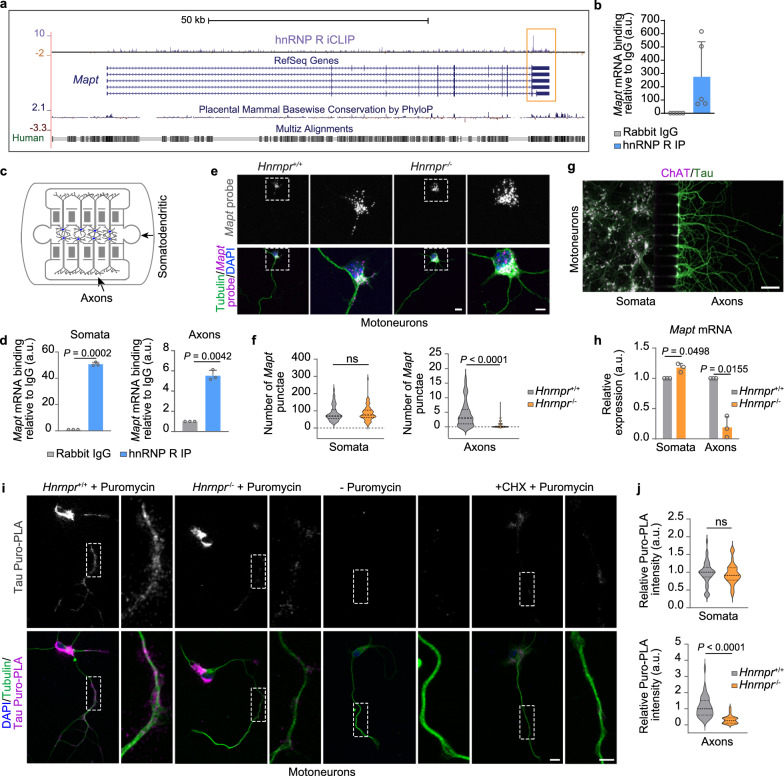
hnRNP R regulates axonal *Mapt* levels and local tau synthesis. **a** UCSC genome browser view showing hnRNP R iCLIP hits along the *Mapt* pre-mRNA. **b** Quantitative PCR (qPCR) of *Mapt* mRNA co-immunoprecipitated with an anti-hnRNP R antibody from DIV 7 mouse motoneurons. Data are mean ± standard deviation (s.d.) of *n* = 5 biological replicates. **c** Schematic of a microfluidic chamber for compartmentalized neuron culture. **d** qPCR analysis of *Mapt* mRNA co-immunoprecipitated with an anti-hnRNP R antibody from compartmentalized DIV 7 mouse hippocampal neurons. Two-tailed one-sample *t*-test. Data are mean ± s.d. of *n* = 3 biological replicates. **e** ViewRNA FISH of *Mapt* mRNA in DIV 5 motoneurons cultured from *Hnrnpr*^+/+^ and *Hnrnpr*^−/−^ mice. Neuronal morphology was visualized by immunostaining for α-tubulin. Scale bars: 10 µm and 5 µm (inset). **f** Number of *Mapt* FISH punctae in somata and axons of *Hnrnpr*^+/+^ and *Hnrnpr*^−/−^ motoneurons. *n* = 73 to 80 motoneurons from four biological replicates. Mann–Whitney test; ns, not significant. **g** DIV 7 motoneurons cultured in a microfluidic chamber and stained for the motoneuron marker choline acetyltransferase (ChAT) and total tau. Scale bar: 100 µm. **h** qPCR analysis of *Mapt* mRNA from somatodendritic and axonal RNA of compartmentalized *Hnrnpr*^+/+^ and *Hnrnpr*^−/−^ motoneurons at DIV 7. Two-tailed one-sample *t*-test. Data are mean ± s.d. of *n* = 3 biological replicates. **i** Puromycin labeling followed by proximity ligation assay (Puro-PLA) for visualization of newly synthesized tau in DIV 6 *Hnrnpr*^+/+^ and *Hnrnpr*.^−/−^ motoneurons. Scale bars: 10 and 5 µm (inset). **j** Tau Puro-PLA intensities in the somata and axons of motoneurons.* n* = 38 to 39 motoneurons from three biological replicates. Unpaired two-tailed Student’s *t*-test (somata) and Mann–Whitney test (axons)

We previously observed that hnRNP R regulates the axonal localization of mRNAs in primary mouse embryonic motoneurons [52]. In line with this function, individual nucleotide resolution crosslinking and immunoprecipitation (iCLIP) has revealed binding of cytosolic hnRNP R to 3’ UTRs of specific mRNAs [52]. To assess whether *Mapt* mRNA is targeted by hnRNP R, we visually inspected the *Mapt* locus and identified an enrichment of hnRNP R iCLIP hits in the 3’ UTR (Fig. 1a). RNA immunoprecipitation using an antibody against hnRNP R confirmed its association with *Mapt* in motoneurons cultured for 7 DIV (Fig. 1b). To investigate the interaction of *Mapt* with hnRNP R in axons, we cultured hippocampal neurons in microfluidic chambers (Fig. 1c). Following RNA immunoprecipitation, we observed hnRNP R binding to *Mapt* in axons (Fig. 1d). This suggests that hnRNP R associates with *Mapt* mRNA and regulates its presence in axons.

To test this possibility, we assessed whether depletion of hnRNP R alters the axonal localization of *Mapt*. For this purpose, we used an hnRNP R-knockout mouse model in which exons 1–5 of the *Hnrnpr* gene were deleted by CRISPR/Cas9-mediated genome engineering [37, 53]. We quantified *Mapt* levels in cultured neurons by FISH using the ViewRNA assay, which utilizes branched-DNA technology to enable signal amplification. This revealed reduced axonal *Mapt* levels in motoneurons cultured from *Hnrnpr*^−/−^ mice relative to motoneurons from *Hnrnpr*^+/+^ mice (Fig. 1e, f). *Mapt* levels in the somata of *Hnrnpr*^−/−^ motoneurons were unchanged (Fig. 1f). Reduction of axonal, but not somatodendritic, *Mapt* mRNA was also detected by qPCR in *Hnrnpr*^−/−^ compared to *Hnrnpr*^+/+^ motoneurons cultured in microfluidic chambers for 7 DIV, a time point at which robust growth of tau-positive axons in the axonal compartment was visible (Fig. 1g, h). We then used Puro-PLA [54] to assess the axonal translation of tau. Results showed significantly reduced tau synthesis in axons but not somata of *Hnrnpr*^−/−^ motoneurons (Fig. 1i, j). In agreement with the FISH and Puro-PLA data, immunostaining revealed lower tau protein levels in axons but not somata of *Hnrnpr*^−/−^ motoneurons (Fig. S1a, b). The effect of hnRNP R depletion on axonal tau levels was more prominent in distal versus proximal axons (Fig. S1b). To evaluate whether axonal microtubules are affected by hnRNP R depletion, we investigated our previously published somatodendritic and axonal proteomics datasets of compartmentalized *Hnrnpr*^−/−^ and *Hnrnpr*^+/+^ motoneurons [37]. We observed no major alterations in tubulin protein levels in axons of hnRNP R-deficient motoneurons (Fig. S1c). Thus, hnRNP R-mediated translocation of the *Mapt* mRNA to axons determines axonal tau protein levels.

### Reduced AD pathology upon loss of hnRNP R

We evaluated the expression of hnRNP R in adult mouse brain by immunohistochemistry. HnRNP R was detectable throughout the forebrain, including cortex and hippocampus, as well as cerebellum and midbrain (Fig. S2). Notably, hnRNP R was particularly abundant in the hippocampus compared to other regions (Fig. S2). Based on our observation that hnRNP R depletion lowers axonal tau levels, we sought to test whether loss of hnRNP R ameliorates pathological alterations occurring in AD brains. For this purpose, we crossed *Hnrnpr*^*−/−*^ with 5×FAD mice that overexpress mutant human amyloid-β precursor protein (APP) and mutant human presenilin-1 harbouring familial AD mutations [55]. We chose 5×FAD mice for several reasons. First, crossing of 5×FAD mice with tau knockout mice improves their plaque pathology [11]. Thus, 5×FAD mice are suitable for assessing beneficial effects of tau depletion. Second, even though 5×FAD mice do not carry a tau transgene or express mutant tau, their brains exhibit pathological forms of tau that are detectable by different phospho-tau antibodies [56–59]. Of note, tau hyperphosphorylation has also been detected in synaptosomes of another mouse model expressing mutant APP and presenilin-1 [60]. Finally, hnRNP R targets the *Mapt* 3’ UTR, which is not contained in commonly used tau transgenic mice such as line PS19 overexpressing mutant tau P301S.

**Fig. 2:**
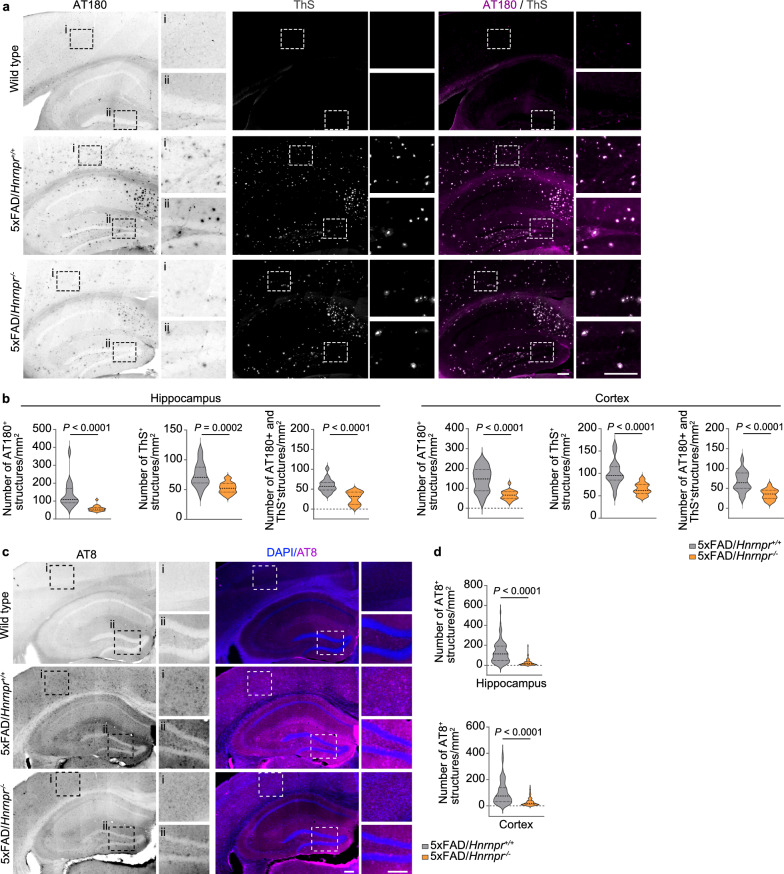
hnRNP R deficiency ameliorates tau pathologies in 5×FAD mice. **a** Immunohistochemical detection of phosphorylated tau using the AT180 antibody and Thioflavin S (ThS) staining in coronal brain sections of 9-month-old wild-type, 5×FAD/*Hnrnpr*^+/+^ and 5×FAD/*Hnrnpr*^−/−^ mice. Boxes indicate magnified regions in cortex (i) and hippocampus (ii). Scale bars: 200 µm. **b** Numbers of AT180^+^, ThS^+^ and AT180^+^ ThS^+^ structures per square millimetre in hippocampus and cortex of 6- and 9-month-old 5×FAD/*Hnrnpr*^+/+^ and 5×FAD/*Hnrnpr*^−/−^ mice. *n* = 16 to 18 brain sections from five to six mice per group. Mann–Whitney test and unpaired two-tailed Student’s *t*-test. **c** Immunohistochemical detection of phosphorylated tau using the AT8 antibody in coronal brain sections of 9-month-old wild-type, 5×FAD/*Hnrnpr*^+/+^ and 5×FAD/*Hnrnpr*^−/−^ mice. Boxes indicate magnified regions in cortex (i) and hippocampus (ii). Scale bars: 200 µm. **d** Number of AT8^+^ structures per square millimetre in hippocampus and cortex of 6- and 9-month-old 5×FAD/*Hnrnpr*^+/+^ and 5×FAD/*Hnrnpr*^−/−^ mice. *n* = 80 to 97 (hippocampus) and 93 to 97 (cortex) brain sections from five to six mice per group. Mann–Whitney test

**Fig. 3:**
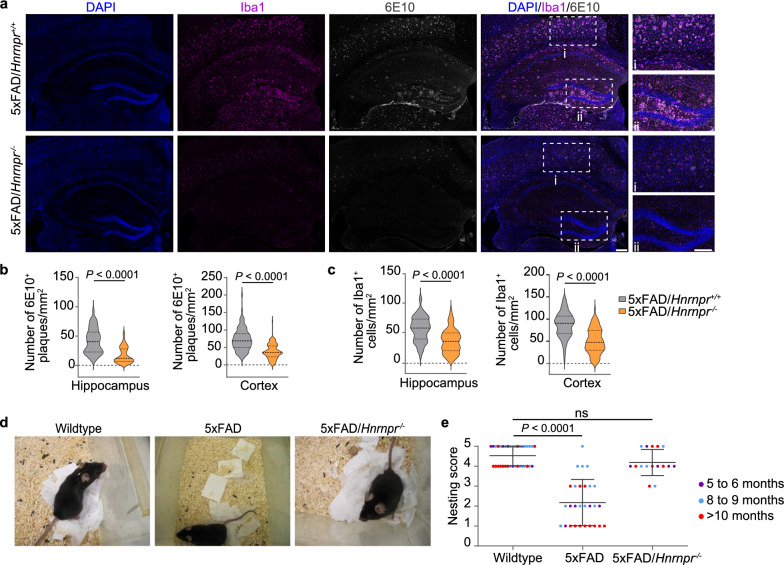
hnRNP R deficiency reduces Aβ plaques in 5×FAD mice. **a** Immunohistochemical detection of microglia using an antibody against Iba1 and of Aβ plaques using the 6E10 antibody in coronal brain sections of 9-month-old 5×FAD/*Hnrnpr*^+/+^ and 5×FAD/*Hnrnpr*^−/−^ mice. Boxes indicate magnified regions in cortex (i) and hippocampus (ii). Scale bars: 200 µm. **b** Number of Aβ plaques per square millimetre in hippocampus and cortex of 6- and 9-month-old 5×FAD/*Hnrnpr*^+/+^ and 5×FAD/*Hnrnpr*^−/−^ mice. *n* = 100 to 105 (hippocampus) and 104 to 121 (cortex) brain sections from five to six mice per group. Mann–Whitney test. **c** Number of Iba1^+^ microglia per square millimeter in hippocampus and cortex of 6- and 9-month-old 5×FAD/*Hnrnpr*^+/+^ and 5×FAD/*Hnrnpr*^−/−^ mice**.** *n* = 100 to 101 (hippocampus) and 100 to 101 (cortex) brain sections from five to six mice per group. Mann–Whitney test. **d** Nesting behaviour of 16-month-old wild-type, 5×FAD and 5×FAD/*Hnrnpr*^−/−^ mice. **e** Nesting scores of 5- to 16-month-old wild-type, 5×FAD and 5×FAD/*Hnrnpr*^−/−^ mice. *n* = 16 to 36 mice per genotype. One-way ANOVA with Holm-Sidak’s multiple comparisons test

Brain sections of 6- and 9-month-old 5×FAD/*Hnrnpr*^+/+^ and 5×FAD/*Hnrnpr*^−/−^ mice were immunostained with the AT180 antibody to detect tau phosphorylated at Thr231 (Fig. 2a). We observed a significant reduction in the number of AT180^+^ aggregates in the hippocampus and cortex of 5×FAD/*Hnrnpr*^−/−^ relative to 5×FAD/*Hnrnpr*^+/+^ mice (Fig. 2a, b). Notably, a large proportion of AT180^+^ aggregates overlapped with accumulations positive for ThS in these brain regions of 5×FAD/*Hnrnpr*^+/+^ mice, which were likewise reduced in 5×FAD/*Hnrnpr*^−/−^ mice (Fig. 2a, b). Similarly, immunostaining with the AT8 antibody that detects tau phosphorylated at Ser202/Thr205 revealed less AT8^+^ aggregates in 5×FAD/*Hnrnpr*^−/−^ mice (Fig. 2c, d). Both AT180^+^ and AT8^+^ structures were absent in the brains of wild-type mice (Fig. 2a, c). Additionally, we visualized Aβ plaques using the 6E10 antibody and observed a significant reduction in the number of Aβ plaques in the hippocampus and cortex of 5×FAD/*Hnrnpr*^−/−^ relative to 5×FAD/*Hnrnpr*^+/+^ mice (Fig. 3a, b). Deposition of Aβ plaques in the brains of 5×FAD mice is accompanied by activation of microglia [61]. In agreement with the reduced plaque burden, we observed reduced microglial activation in the hippocampus and cortex of 5×FAD/*Hnrnpr*^−/−^ mice by immunostaining for the microglial marker Iba1 (Fig. 3a, c). To investigate the functional effects of hnRNP R depletion in 5×FAD mice, we assessed nest building, which is commonly used to monitor cognitive and social abilities [50]. Nest building activity is hippocampus-dependent and reduced in 5×FAD mice [62, 63]. We assessed nesting activity of wild-type, 5×FAD and 5×FAD/*Hnrnpr*^−/−^ mice aged 5 to 16 months and observed that deletion of hnRNP R restored nesting behaviour of 5×FAD mice (Fig. 3d, e). Depletion of hnRNP R thus ameliorates both tau and amyloid pathology in 5×FAD mice and functionally improves their phenotype.

### ASOs for lowering axonal *Mapt* mRNA levels

**Fig. 4:**
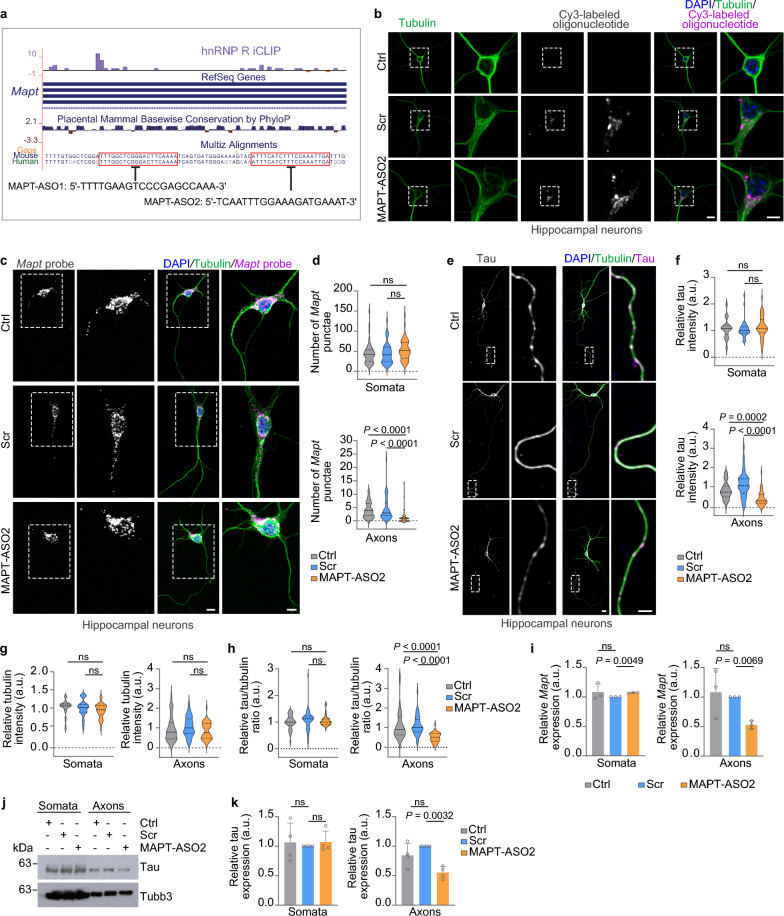
MAPT-ASO treatment reduces axonal tau levels. **a** Design of two antisense oligonucleotides (MAPT-ASO1 and MAPT-ASO2) for blocking the interaction between hnRNP R and *Mapt* mRNA. **b** Immunofluorescence imaging of DIV 25 untreated (Ctrl) mouse hippocampal neurons and hippocampal neurons treated with 10 µmol/L of a Cy3-labeled scramble oligonucleotide or MAPT-ASO2. Scale bars: 10 and 5 µm (inset). Images are representative of at least three biological replicates. **c** *Mapt* FISH of DIV 6 untreated (Ctrl) mouse hippocampal neurons and hippocampal neurons treated with scramble oligonucleotide or MAPT-ASO2. Scale bars: 10 and 5 µm (inset). **d** Number of *Mapt* FISH punctae in the somata and axons of hippocampal neurons. *n* = 42 to 47 hippocampal neurons from three biological replicates. Kruskal Wallis with Dunn’s multiple comparisons test. **e** Total tau and α-tubulin immunostaining of DIV 25 untreated (Ctrl) mouse hippocampal neurons and hippocampal neurons treated with scramble oligonucleotide or MAPT-ASO2, with distal regions of the axon marked. Scale bars: 10 and 5 µm (inset). **f–h** Immunointensities of tau (**f**) and α-tubulin (**g**) and tau/tubulin ratios (**h**) in the somata and distal axonal regions of hippocampal neurons. *n* = 38 to 42 hippocampal neurons from three biological replicates. Kruskal Wallis with Dunn’s multiple comparisons test. **i** qPCR analysis of *Mapt* mRNA from somatodendritic and axonal RNA of compartmentalized DIV 7 cortical neurons treated with scramble oligonucleotide or MAPT-ASO2. Two-tailed one-sample *t*-test. Data are mean ± s.d. of *n* = 3 biological replicates. **j** Immunoblotting for total tau (Tau46 antibody) from somatodendritic and axonal lysates of compartmentalized DIV 7 cortical neurons treated with scramble oligonucleotide or MAPT-ASO2. Uncropped immunoblot images are shown in Additional file 3. **k** Immunoblotting intensities of total tau from somatodendritic and axonal lysates of compartmentalized cortical neurons (**j**). Two-tailed one-sample *t*-test. Data are mean ± s.d. of *n* = 4 biological replicates

Having shown that hnRNP R loss reduces axonal tau and lowers plaque and phospho-tau deposition, we next sought to achieve axonal tau reduction more selectively through blocking the association between hnRNP R and *Mapt*. For this purpose, we designed two ASOs (MAPT-ASO1 and 2) complementary to the *Mapt* 3’ UTR regions with hnRNP R iCLIP hits (Fig. 4a). The MAPT-ASOs contained a phosphorothioate backbone and 2’-*O*-methyl ribose modifications at all positions to prevent *Mapt* mRNA degradation. We selected binding regions that were conserved between mice and humans. For initial analysis of uptake conditions, a Cy3-labeled sense oligonucleotide was used. Efficient uptake was observed by incubating mouse motoneurons or hippocampal neurons with 10 µmol/L oligonucleotide (Fig. S3a, b), similar to a previous study [64]. Motoneurons treated with MAPT-ASO1 or 2 and cultured for 6 DIV showed reduced axonal *Mapt* mRNA levels compared to untreated motoneurons as detected by FISH (Fig. S3c, d). A similar reduction in axonal *Mapt* was also detectable in cultured hippocampal neurons (Fig. S3e, f). Importantly, *Mapt* levels in the cell bodies of MAPT-ASO1- and 2-treated neurons were unchanged (Fig. S3c–f).

Next, we investigated whether MAPT-ASO-mediated depletion of axonal *Mapt* can also downregulate tau protein in axons. Given the long half-life of tau [65, 66], we assessed tau levels by immunostaining in MAPT-ASO-treated as well as untreated motoneurons and hippocampal neurons cultured for 11 and 22–25 DIV, respectively. Following MAPT-ASO treatment, tau levels were reduced in axons but not cell bodies of motoneurons and hippocampal neurons (Fig. S4a-d). The axonal tau reduction was more pronounced for MAPT-ASO2 compared to MAPT-ASO1 (Fig. S4b, d). Together, these data indicate that tau protein levels can be selectively reduced in axons by ASOs blocking the hnRNP R-binding sites in the *Mapt* 3’ UTR.

### MAPT-ASO2 reduces axonal tau

Since MAPT-ASO2 reduced axonal tau more efficiently than MAPT-ASO1, we used MAPT-ASO2 and compared it to a scrambled version of it (Scr) as an additional control. At 10 µmol/L, Cy3-labeled MAPT-ASO2 and Scr were efficiently taken up by motoneurons (Fig. S5a) and hippocampal neurons (Fig. 4b). Compared to Scr, MAPT-ASO2 significantly downregulated *Mapt* levels in axons but not somata of motoneurons (Fig. S5b, c) and hippocampal neurons (Fig. 4c, d). Likewise, tau protein levels were reduced in axons of MAPT-ASO2-treated motoneurons (Fig. S5d, e) and hippocampal neurons (Fig. 4e, f) compared to Scr-treated neurons. In contrast, tau levels in the somata of MAPT-ASO2-treated neurons remained unchanged (Fig. 4e, f, Fig. S5d, e). Furthermore, we observed that the levels of axonal α-tubulin were unaffected by MAPT-ASO2 treatment, indicating that axonal microtubules are not altered by axonal tau depletion (Fig. 4g, h). To substantiate our findings from immunostainings, we quantified the somatodendritic and axonal *Mapt* mRNA and tau protein levels in compartmentalized cortical neurons treated with Scr or MAPT-ASO2 by qPCR and immunoblotting, respectively. Consistent with the FISH and immunostaining data, the axonal levels of *Mapt* mRNA and tau protein were significantly reduced upon MAPT-ASO2 treatment, while their levels in cell bodies were unchanged (Fig. 4i–k). In contrast, the axonal level of the β-actin-encoding *Actb* mRNA, the 3’ UTR of which also interacts with hnRNP R for axonal localization [67, 68], was not reduced by MAPT-ASO2 treatment (Fig. S6a), which provides evidence for the specificity of MAPT-ASO2. Additionally, to assess whether MAPT-ASO2 treatment affects the subcellular distribution of hnRNP R, we performed hnRNP R immunostaining and observed no changes in the levels of hnRNP R in somata and axons of MAPT-ASO2-treated hippocampal neurons (Fig. S6b, c).

**Fig. 5:**
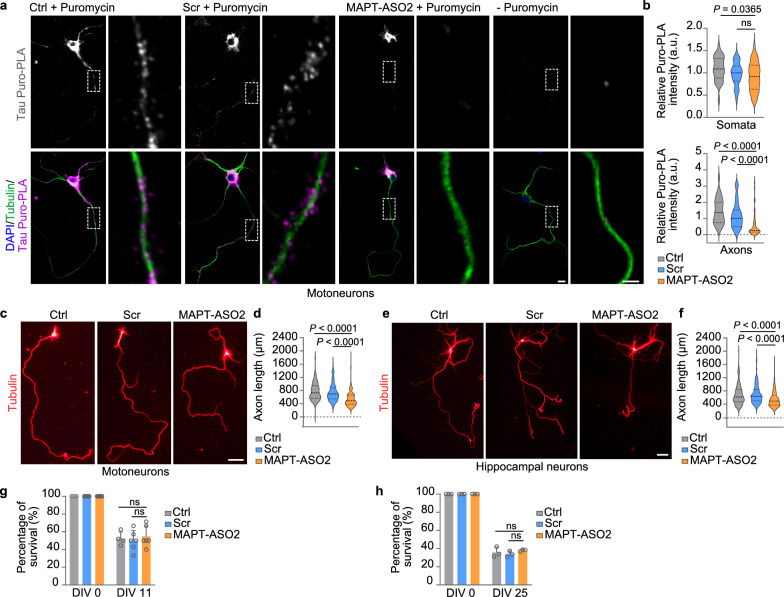
Axonal tau reduction with MAPT-ASO2 phenocopies tau deficiency. **a** Tau Puro-PLA in DIV 6 untreated (Ctrl) mouse motoneurons and motoneurons treated with scramble oligonucleotide or MAPT-ASO2. Scale bars: 10 µm and 5 µm (inset). **b** Tau Puro-PLA intensities in the somata and axons of motoneurons.* n* = 30 to 44 motoneurons from three biological replicates. Kruskal Wallis with Dunn’s multiple comparisons test. **c** Morphology of DIV 7 untreated (Ctrl) mouse motoneurons and motoneurons treated with scramble oligonucleotide or MAPT-ASO2. Scale bar: 50 µm. **d** Axon lengths of motoneurons. *n* = 154 to 201 motoneurons from three biological replicates. Kruskal Wallis with Dunn’s multiple comparisons test. **e** Morphology of DIV 25 untreated (Ctrl) mouse hippocampal neurons and hippocampal neurons treated with scramble oligonucleotide or MAPT-ASO2. Scale bar: 50 µm. **f** Axon lengths of hippocampal neurons. *n* = 201 to 216 hippocampal neurons from three biological replicates. Kruskal Wallis with Dunn’s multiple comparisons test. **g** Quantification of motoneuron survival on DIV 11. Two-way ANOVA with Tukey’s multiple comparisons test. Data are mean ± s.d. of *n* = 4 to 6 biological replicates. **h** Quantification of hippocampal neuron survival on DIV 25. Two-way ANOVA with Tukey’s multiple comparisons test. Data are mean ± s.d. of *n* = 3 biological replicates

Motoneurons treated with MAPT-ASO2 revealed a reduced Puro-PLA signal for tau in axons compared to Scr-treated and untreated motoneurons (Fig. 5a, b). Importantly, tau synthesis in cell bodies was unaffected by MAPT-ASO2 relative to Scr treatment (Fig. 5a, b). Thus, MAPT-ASO2 treatment can reduce axonal levels of *Mapt*, resulting in less axonal tau due to lowered local translation. In agreement with reduced axon growth observed in tau-depleted neurons [69, 70], axon lengths of motoneurons and hippocampal neurons treated with MAPT-ASO2 were reduced compared to Scr treatment (Fig. 5c-f). Survival of motoneurons and hippocampal neurons was unaffected by MAPT-ASO2 treatment (Fig. 5g, h). To assess neuronal functionality, the spontaneous activity of cultured hippocampal neurons treated with MAPT-ASO2 was investigated in calcium imaging experiments. As a result, we observed no statistically significant alterations in frequencies and mean amplitudes of calcium transients between untreated, Scr-treated and MAPT-ASO2-treated neurons (Fig. S7a-c). Thus, general neuronal excitability as a measure of neuronal health is not affected by MAPT-ASO2 treatment.

Next, we designed additional MAPT-ASOs along the *Mapt* 3’ UTR in regions that contain hnRNP R iCLIP hits and that are conserved between mouse and human (Fig. S8a, b). We screened these ASOs by FISH in hippocampal neurons for their potential to reduce axonal *Mapt* mRNA levels and identified several candidates that lowered *Mapt* levels to less than 50% relative to untreated hippocampal neurons (Fig. S8b, c). Of these, MAPT-ASO5 overlapped the binding site of MAPT-ASO1, and MAPT-ASO19 and 20 were shortened versions of MAPT-ASO2 with a length of 18 and 16 nucleotides, respectively (Fig. S8a, b). Treatment of hippocampal neurons with MAPT-ASO5 or 20 reduced axonal levels of *Mapt* mRNA and tau protein as well as axon growth similar to treatment with MAPT-ASO1 and 2 (Fig. S9a-e). Taken together, our findings indicate that the *Mapt* 3’ UTR can be targeted by blocking ASOs to reduce tau levels locally in axons.

### MAPT-ASO2-mediated reduction of axonal tau in iPSC-derived human neurons

We then tested MAPT-ASO2 for its efficacy in reducing axonal tau in human neurons. For this purpose, we differentiated iPSCs into cholinergic human motoneurons using small molecules and cultured them for 25 DIV [41] (Fig. S10a). At this time point, Piccolo (PCLO), the expression of which correlates with functional neuronal maturity [71], was detectable by immunostaining (Fig. S10a). We first tested ASO uptake by treating human motoneurons on DIV 4 with 10 µmol/L of the Cy3-labeled Scr ASO. At this concentration, the Scr ASO was detectable in motoneurons cultured for 21 DIV after ASO treatment (Fig. S10b). Next, the motoneurons were treated with 10 µmol/L MAPT-ASO2 or Scr, or left untreated. In distal axons, treatment with MAPT-ASO2 significantly reduced axonal tau levels compared to untreated and Scr-treated motoneurons (Fig. S10c, d). While the reduction in tau in proximal axonal regions was not significant between MAPT-ASO2- and untreated neurons, it reached significance when compared to Scr-treatment (Fig. S10c, d). In contrast, tau levels in the somata of MAPT-ASO2-treated motoneurons were unchanged (Fig. S10c, d). Of note, we observed a reduction in the levels of axonal α-tubulin in the MAPT-ASO2-treated human motoneurons (Fig. S10d), indicating that they respond differently compared to hippocampal neurons, in which axonal α-tubulin levels were unaffected by MAPT-ASO2 treatment (Fig. 4g, h). Nevertheless, our data show that MAPT-ASO2 has similar high efficacy in lowering axonal tau in human neurons compared to mouse neurons.

### Intracerebroventricular administration of MAPT-ASO2 reduces tau and amyloid deposition in 5×FAD mice

5×FAD mice develop an AD-like pathology at young ages, with Aβ plaques being present in the brain as early as 2 months and phospho-tau aggregates detectable at 5–6 months of age [55, 56]. To evaluate whether MAPT-ASO2 treatment can reverse these pathologies, MAPT-ASO2 or vehicle control (aCSF) was administered intracerebroventricularly in 8-month-old female 5×FAD mice via Alzet minipumps. We only used female 5×FAD mice because they exhibit a more robust pathology compared to males [61, 72]. The Alzet minipump allowed continuous infusion of MAPT-ASO2 over 42 days at a rate of 1.8 µg/day (total 75.6 µg). Twenty 5×FAD mice were treated with aCSF and 17 treated with MAPT-ASO2. In total, 11 animals died prematurely or had to be euthanized due to reduced general health condition, including 8 from the aCSF-treated group and 3 from the MAPT-ASO2-treated group. This result indicates that the premature deaths cannot be attributed to the treatment with ASOs. Consistently, there was no difference in body weight between MAPT-ASO2-injected and aCSF-injected 5×FAD mice (Fig. S11a).

**Fig. 6:**
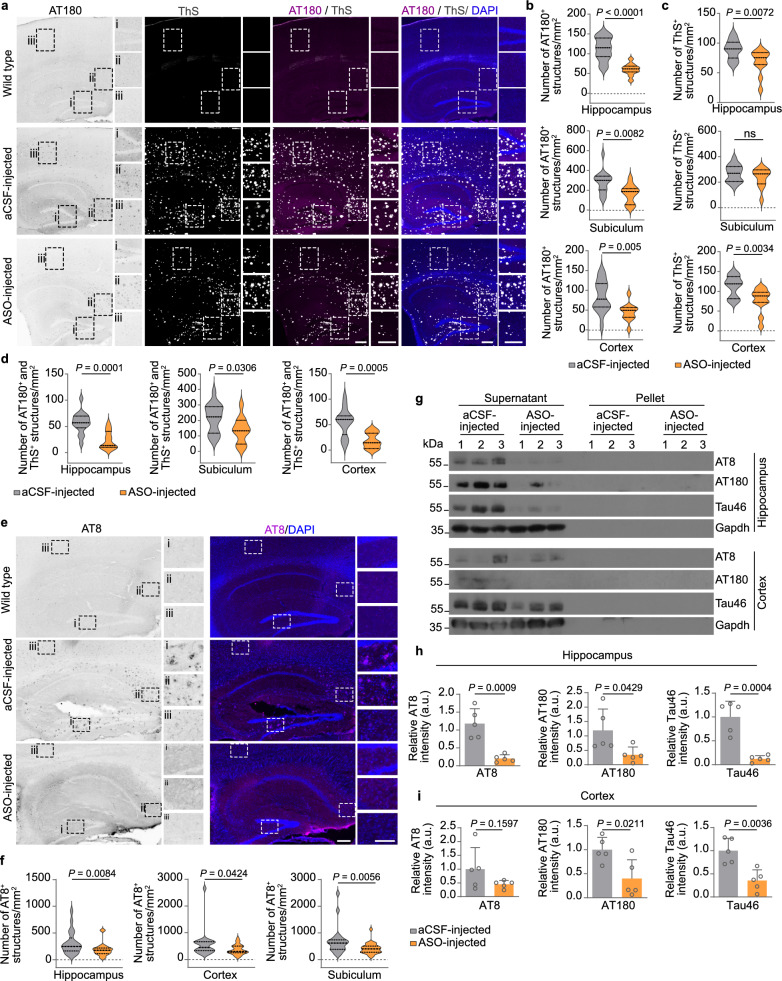
Intracerebroventricular delivery of MAPT-ASO2 ameliorates tau pathology in 5×FAD mice. **a** Immunohistochemical detection of phosphorylated tau using the AT180 antibody in sagittal brain sections of wild-type, aCSF- and MAPT-ASO2-treated 5×FAD mice. Boxes indicate magnified regions in hippocampus (i), subiculum (ii) and cortex (iii). Scale bars: 200 and 100 µm (inset). **b–d** Numbers of AT180^+^ (**b**), ThS^+^ (**c**) and AT180^+^ ThS^+^ (**d**) structures per square millimetre in hippocampus, subiculum and cortex. *n* = 11 to 16 brain sections from three mice per group. Unpaired two-tailed Student’s *t*-test. **e** Immunohistochemical detection of phosphorylated tau using the AT8 antibody in sagittal brain sections of aCSF- and MAPT-ASO2-treated 5×FAD mice. Boxes indicate magnified regions in hippocampus (i), subiculum (ii) and cortex (iii). Scale bars: 200 and 100 µm (inset). **f** Number of AT8^+^ structures per square millimetre in hippocampus, subiculum and cortex. *n* = 21 to 26 brain sections from five mice per group. Mann–Whitney test. **g** Immunoblotting for total tau (Tau46 antibody) and phospho-tau (AT8 and AT180 antibodies) present in total protein lysates from hippocampus and cortex of aCSF- and MAPT-ASO2-treated 5×FAD mice. Uncropped immunoblot images are shown in Additional file 3. **h, i** Immunoblotting intensities of total tau and phospho-tau for lysates from hippocampus (**h**) and cortex (**i**). *n* = 5 biological replicates. Unpaired two-tailed Student’s *t*-test

**Fig. 7:**
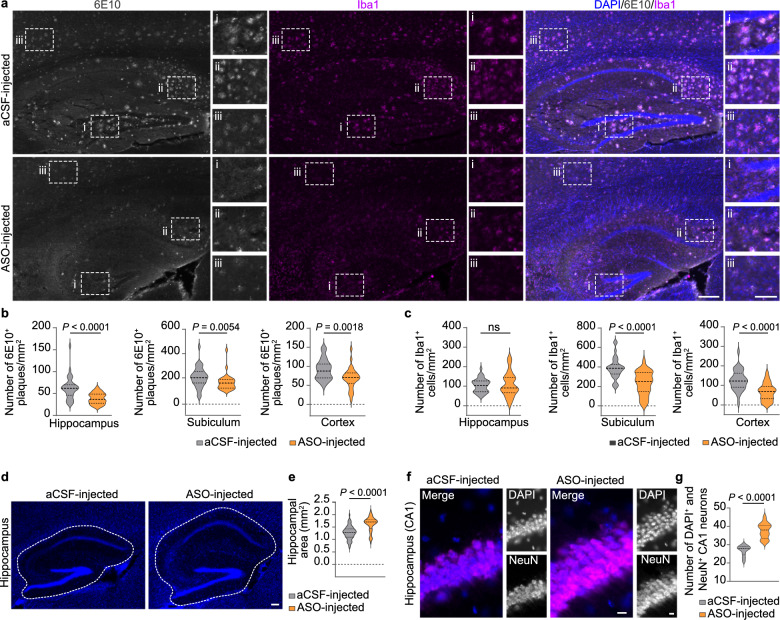
Reduced Aβ pathology in 5×FAD mice treated with MAPT-ASO2. **a** Immunohistochemical detection of microglia using an antibody against Iba1 and of Aβ plaques using the 6E10 antibody in sagittal brain sections of 5×FAD mice treated with aCSF or MAPT-ASO2. Boxes indicate magnified regions in hippocampus (i), subiculum (ii) and cortex (iii). Scale bars: 200 and 100 µm (inset). **b, c** Numbers of Aβ plaques (**b**) and Iba1^+^ microglia (**c**) per square millimetre in hippocampus, subiculum and cortex. *n* = 29 to 33 brain sections from five mice per group. Mann–Whitney and unpaired two-tailed Student’s *t*-test. **d** DAPI-stained brain sections of aCSF- and MAPT-ASO2-injected 5×FAD mice. Hippocampal regions are marked by dotted lines. Scale bar: 100 µm. **e** Area of hippocampal regions. *n* = 46 to 53 brain sections from five mice per group. Mann–Whitney test. **f** Immunohistochemical detection of NeuN in hippocampal CA1 regions of brain sections of 5×FAD mice treated with aCSF or MAPT-ASO2. Scale bars: 10 µm. **g** Number of DAPI^+^ NeuN^+^ CA1 neurons per CA1 region shown in (**f**). *n* = 10 to 13 brain sections from three mice per group. Unpaired two-tailed Student’s *t*-test

Eight weeks following Alzet pump implantation, mouse brains were collected for histochemical analysis. FISH analysis using an LNA-modified Cy3-labeled sense oligonucleotide complementary to MAPT-ASO2 revealed widespread diffusion of MAPT-ASO2 throughout the brain whereas no FISH signal was detectable in the brains of aCSF-treated mice (Fig. S11b). AT180 immunostaining showed significantly less AT180^+^ aggregates in the hippocampus and cortex of MAPT-ASO2-treated mice (Fig. 6a, b). Notably, MAPT-ASO2 treatment also led to a reduced number of AT180^+^ aggregates in the subiculum, in which Aβ plaques are particularly prominent and appear early in 5×FAD mice (Fig. 6b) [55]. Additionally, the number of ThS^+^ structures, a large proportion of which were AT180^+^, was significantly reduced in these brain regions in MAPT-ASO2-treated mice (Fig. 6a, c, d). Consistent with these results, we observed a significant reduction of AT8^+^ aggregates in the hippocampus, subiculum, and cortex of MAPT-ASO2-treated mice (Fig. 6e, f). Both AT180^+^ and AT8^+^ aggregates were not detectable in the brains of wild-type mice (Fig. 6a, e). To corroborate these findings, we extracted total proteins from hippocampus and cortex for fractionation into supernatant and pellet to assess the levels of total and phosphorylated tau by immunoblotting (Fig. 6g–i). In agreement with a previous study [73], tau was predominantly present in the supernatant (Fig. 6g). Both total tau detected with the Tau46 antibody and phospho-tau detected with the AT8 and AT180 antibodies were significantly reduced in the hippocampus of 5×FAD mice treated with MAPT-ASO2 (Fig. 6h). In the cortex, total tau and phospho-tau detected with the AT180 antibody were significantly reduced in MAPT-ASO2-treated mice while phospho-tau detected with the AT8 antibody showed a tendency towards reduction (Fig. 6i). It has previously been shown that reduction of tau pathology in AD mouse models also lowers their Aβ plaque burden [11, 74]. Therefore, we assessed the deposition of Aβ plaques and the presence of activated microglia in brains of aCSF- and MAPT-ASO2-treated mice by 6E10 and anti-Iba1 immunostaining (Fig. 7a). The number of Aβ plaques was significantly reduced in hippocampus, subiculum and cortex (Fig. 7b). This was accompanied by significantly reduced numbers of Iba1^+^ microglia in subiculum and cortex (Fig. 7c). We also observed that the hippocampus of MAPT-ASO2-injected 5×FAD mice was larger in size and contained a higher number of NeuN^+^ neurons per CA1 region compared to aCSF-treated 5×FAD mice (Fig. 7d-g). Thus, administration of MAPT-ASO2 reduces tau and amyloid pathology, and prevents neurodegeneration in 5×FAD mice.

**Fig. 8:**
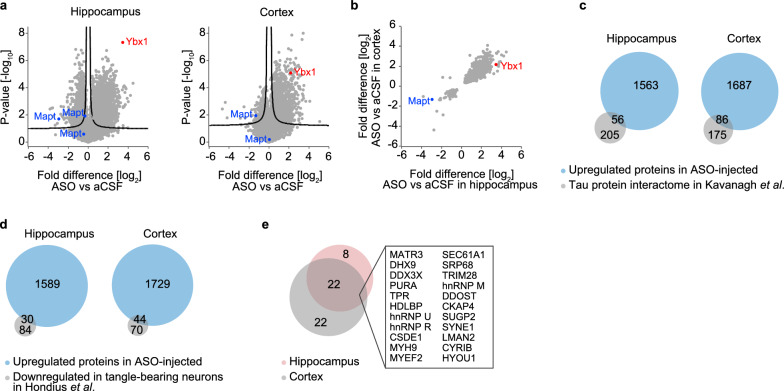
Proteomic analysis of brains of MAPT-ASO2-treated 5×FAD mice. **a** Volcano plots of differential protein expression in hippocampus and cortex of ASO-injected relative to aCSF-injected 5×FAD mice. Note that tau was detected as multiple isoforms. *n* = 4 to 5 biological replicates. **b** Scatter plots depicting fold differences of significant (*P*_adj_ < 0.05) protein changes in hippocampus and cortex of ASO-injected relative to aCSF-injected 5×FAD mice. **c** Comparison of proteins significantly (*P* < 0.05) upregulated in hippocampus and cortex of ASO-injected relative to aCSF-injected 5×FAD mice with tau-interacting proteins described by Kavanagh et al. [75] **d** Comparison of proteins significantly (*P* < 0.05) upregulated in hippocampus and cortex of ASO-injected relative to aCSF-injected 5×FAD mice with proteins downregulated in tangle-bearing neurons in post-mortem AD brains identified by Hondius et al. [76]. **e** Overlay of proteins rescued by MAPT-ASO2 treatment in hippocampus and cortex

We performed mass spectrometry analysis on supernatants of fractionated hippocampal and cortical lysates in order to identify proteins altered in brains of 5×FAD mice in response to MAPT-ASO2 treatment (Table S2). In agreement with the immunoblot analysis (Fig. 6h, i), we observed reduced tau levels in both tissues following MAPT-ASO2 administration (Fig. 8a). The levels of tubulin proteins were largely unaffected by MAPT-ASO2 treatment (Fig. S12). Across all proteins, the magnitude of fold difference was higher for upregulated than for downregulated proteins. The RNA-binding protein Ybx1 was the most significantly upregulated protein in hippocampal lysate, and was also highly upregulated in cortex (Fig. 8a). Overall, there was a strong correlation between proteins significantly altered in hippocampus and cortex of 5×FAD mice upon MAPT-ASO2 treatment (Fig. 8b). Comparison of upregulated proteins with 261 proteins identified as tau interactors across several tau interactome studies [75] revealed that 56 and 86 of the proteins upregulated in hippocampus and cortex, respectively, of treated 5×FAD mice are associated with tau, suggesting that these proteins are stabilized upon tau depletion (Fig. 8c). We also compared proteins upregulated by MAPT-ASO2 treatment with proteins downregulated in tangle-bearing neurons isolated from human post-mortem AD hippocampus [76]. This revealed a strong overlap between these datasets indicating that MAPT-ASO2 treatment can at least partially restore the levels of proteins that are reduced by tau aggregation (Fig. 8d). Among proteins whose levels were corrected by MAPT-ASO2 treatment were the nuclear basket protein TPR and several RNA-processing proteins such as the RNA helicases DHX9 and DDX3X, and the RNA-binding proteins MATR3, PURA, hnRNP U, hnRNP R and hnRNP M (Fig. 8e).

## Discussion

Tau aggregation has emerged as a central pathological trigger in AD, and axonal tau deposition has been identified as an early event in AD pathogenesis, preceding tau tangle formation in the somata. We identified a mechanism by which the RNA-binding protein hnRNP R localizes *Mapt* mRNA in axons for local synthesis of tau. Inhibition of this process by depleting hnRNP R or blocking its interaction with the 3’ UTR of *Mapt* with ASOs lowers axonal tau levels and ameliorates tau and Aβ deposition in brains of 5×FAD mice. Our data thus support the use of axonal tau-lowering ASOs as a therapeutic strategy for treatment of AD and other tauopathies.

Multiple lines of evidence have revealed a reciprocal relationship between tau tangles and Aβ plaques. Tau pathology can be induced in wild-type mice by intracerebral administration of tau fibrils from AD brains [77]. Injection of AD tau fibrils into the brains of 5×FAD mice containing Aβ plaques enhanced this effect, leading to more pronounced phospho-tau aggregation [73]. Importantly, following AD tau fibril injection, axonal tau aggregation preceded the formation of phospho-tau aggregates in neuronal somata [73]. These findings indicate that Aβ plaques can drive tau pathology. Vice versa, results from tau knockout mice have shown reduced Aβ plaque deposition and improvement of Aβ-induced memory defects upon removal of tau [11, 12, 74]. Furthermore, carriers with the presenilin-1 (*PSEN1*) E280A mutation, normally causing autosomal-dominant AD, have been identified that are spared from early-onset AD due to rare protective mutations in *APOE3* or *RELN* [78, 79]. Importantly, these cases showed high plaque burden but reduced tau pathology, supporting the notion that tau deposition is causative in AD.

While neurofibrillary tangles are a major pathological hallmark in brains of AD patients, there is accumulating evidence that prefibrillar tau oligomers, particularly at synaptic sites, rather than mature tangles, confer neurotoxicity. Transgenic mice expressing mutant human P301L tau exhibit progressive neuronal loss that ceases upon transgene suppression, despite ongoing tangle deposition [80]. Hippocampal injection of tau oligomers, but not fibrils, impaired memory function and reduced levels of synaptic proteins [81]. Non-demented AD cases show reduced tau oligomers at synapses [82]. Finally, oligomeric tau accumulates at synapses prior to tau tangle deposition and can spread transsynaptically [23, 83]. While 5×FAD mice do not develop tau tangles per se [73], they exhibit pathological tau phosphorylation [56], which was reduced by treatment with MAPT-ASO2 in our study. In conjunction with the finding that tau depletion rescues neuronal loss and memory defects [11], this indicates that 5×FAD mice are suitable for studying tauopathy mechanisms. In agreement with this notion, we observed that the levels of many proteins reduced in tangle-bearing neurons in human AD brains could be restored in the brains of 5×FAD mice by MAPT-ASO2 treatment. Conspicuously, we identified several RNA-binding proteins whose levels were upregulated upon MAPT-ASO2 injection. These changes might be indicative of restored RNA metabolism, as RNA-binding proteins have been shown to aggregate as a consequence of tau pathology [84]. Furthermore, we detected upregulated levels of the nuclear basket protein TPR in brains of MAPT-ASO2-treated 5×FAD mice. Pathological tau has previously been shown to impair nucleocytoplasmic transport [85] and it is thus possible that the MAPT-ASO2-induced increase in TPR levels alleviates this defect. Overall, we identified Ybx1 as the protein most strongly upregulated by MAPT-ASO2 treatment in brains of 5×FAD mice. Ybx1 associates with tau and, interestingly, we also identified it as a top interactor of hnRNP R previously [75, 86]. Treatment of 5×FAD mice with Ybx1 inhibited Aβ plaque deposition, further signifying its modifying role in disease progression [87]. Together, our results indicate that ASO-mediated targeting of axonal tau can mitigate protein alterations induced by tau and plaque pathologies in 5×FAD mice.

Based on its causal role in AD pathology, tau reduction has emerged as a promising therapeutic approach for treatment of AD and other tauopathies. Several strategies are currently being developed to lower tau levels. Immunotherapies utilize antibodies against tau to induce clearance of tau tangles. However, to be efficacious, tau aggregates need to be cleared intracellularly, which might be challenging to achieve with tau immunotherapies [88]. Additionally, the existence of multiple tau splice isoforms and oligomeric tau species requires careful identification of the most promising tau form to be targeted [89]. Finally, immunotherapies might be associated with increased risks for adverse effects such as inflammatory responses, cerebral edemas or hemorrhages. An alternative might be the depletion of tau through ASO-mediated degradation of the *MAPT* mRNA. This approach can lower tau levels in the nervous system and ameliorate symptoms in PS19 tauopathy mice [17]. However, chronic depletion of tau throughout neurons might be detrimental at old age due to absence of compensatory mechanisms that exist at young and middle ages [19]. Removing tau acutely in adult mice also causes cognitive deficits due to lack of such developmental compensation [20]. In addition, tau exerts functions in neuronal cell bodies, such as DNA damage protection [90], and these functions might be affected by complete tau removal. Thus, the development of more targeted approaches for tau reduction selectively in axons while sparing physiological functions of tau in neuronal cell bodies might prevent the occurrence of side effects associated with global tau depletion. In AD, the production rate of tau is increased and correlates with Aβ plaque deposition [66]. It remains to be determined to what extent this effect is due to enhanced local tau synthesis in axons and whether such an increase can be effectively targeted by MAPT-ASOs.

The strategy outlined here makes use of the hnRNP R-dependent axonal transport of *Mapt* mRNA and its local translation into tau protein. Depletion of hnRNP R in 5×FAD mice or treatment with MAPT-ASO2 reduced both phospho-tau aggregates and Aβ plaques. Importantly, we detected reduced levels of tau phosphorylated at Thr231 with the AT180 antibody and at Ser202/Thr205 with the AT8 antibody. Phosphorylation of tau at Thr231 is considered an early event during tau pathogenesis, which abrogates the ability of tau to bind to microtubules [91]. In contrast, phosphorylation at Ser202/Thr205 occurs at later stages and correlates with tau fibrillization [92]. Thus, axonal tau reduction effectively inhibits the series of tau phosphorylation events already at an early stage. By blocking the interaction between *Mapt* and hnRNP R with ASOs, a reduction in axonal tau levels can be achieved without affecting tau levels in the somatodendritic compartment, which we confirmed in cultured mouse and human neurons. While we characterized MAPT-ASO2 in detail, other ASOs described here might be equally efficient for lowering axonal tau. Interestingly, the efficacies of MAPT-ASO19 and 20 and of MAPT-ASO5 were comparable to that of MAPT-ASO2. Since 19 and 20 are shorter versions of MAPT-ASO2, and MAPT-ASO5 binds in close vicinity to MAPT-ASO2, this finding points towards this particular *MAPT* 3’ UTR region as the most promising target sequence.

In the brains of 5×FAD mice injected with MAPT-ASO2, we observed a strong reduction of total tau. While we cannot rule out that MAPT-ASO2 reduces tau in neuronal cell bodies if administered over prolonged periods of time, this finding might reflect the predominant presence of tau in axons in the brain, particularly at distal axonal endings [93, 94]. Importantly, we observed reduced numbers of phospho-tau aggregates and Aβ plaques in 5×FAD mice treated with MAPT-ASO2, indicating that targeting axonal tau is sufficient for alleviating AD pathology. In future studies, a detailed behavioural analysis of MAPT-ASO2-treated 5×FAD mice or humanized tauopathy mice [95] needs to be conducted to assess the effects of MAPT-ASO2 administration on cognitive parameters altered by amyloid and tau pathologies.

## Conclusion

Together, our findings represent a first step towards more selective tau-targeting strategies. If administered sufficiently early in the course of the disease, the MAPT-ASOs described here might be useful for limiting the initiation and spreading of tau pathology in AD in a more targeted manner compared to approaches aimed at global tau depletion. Either alone or in combination with recently developed therapies targeting Aβ plaques [96], axonal tau-targeting MAPT-ASOs might thus hold the potential to slow down AD progression.

## Supplementary Information

Additional file 1 (PDF 7263 KB) **Table S1**. Antibodies and primers. **Fig. S1**. hnRNP R regulates axonal tau levels. **Fig. S2**. hnRNP R is expressed in various regions of mouse brain. **Fig. S3**. Comparison of MAPT-ASO1 and -ASO2 treatment for reduction of axonal *Mapt *mRNA levels. **Fig. S4**. Comparison of MAPT-ASO1 and -ASO2 treatment for reduction of axonal tau levels. **Fig. S5**. Treatment with MAPT-ASO2 reduces axonal tau levels in motoneurons. **Fig. S6**. MAPT-ASO2 treatment has no effect on axonal hnRNP R level. **Fig. S7**. Spontaneous calcium activity of hippocampal neurons reveals no differences between control, scr-treated and MAPT-ASO2-treated cells. **Fig. S8**. Screening of additional ASOs for reducing axonal Mapt mRNA levels. **Fig. S9**. Validation of additional MAPT-ASOs for axonal tau reduction. **Fig. S10**. MAPT-ASO2-mediated reduction of axonal tau in iPSC-derived human motoneurons. **Fig. S11**. Body weight of aCSF- and ASO-injected 5×FAD mice. **Fig. S12**. Tubulin proteins are largely unaffected by MAPT-ASO2 treatment.

Additional file 2 (XLSX 6339 KB) Table S2. Raw data of mass spectrometry analysis.

Additional file 3 (PDF 91 KB) Uncropped immunoblot images.

## Data Availability

The raw mass spectrometry proteomics data have been deposited to the ProteomeXchange Consortium via the PRIDE partner repository [97] with the dataset identifier PXD053387.

## Abbreviations

Aβ: Amyloid-β
aCSF: Artificial cerebrospinal fluid
AD: Alzheimer’s disease
ASO: Antisense oligonucleotide
BDNF: Brain-derived neurotrophic factor
DAPI: 4′,6-Diamidino-2-phenylindole
DPBS: Dulbecco’s Phosphate Buffered Saline
FISH: Fluorescence in situ hybridization
hnRNP R: Heterogeneous nuclear ribonucleoprotein R
iPSC: Induced pluripotent stem cells
LNA: Locked nucleic acid
NPC: Neural precursor cells
PLA: Proximity ligation assay
PMA: Purmorphamine
qPCR: Quantitative PCR
TFA: Trifluoroacetic acid
