# Supplementary material for: Axonal tau reduction ameliorates tau and amyloid pathology in a mouse model of Alzheimer’s disease
Source: Transl Neurodegener. 2025 Jul 29;14:39. doi: 10.1186/s40035-025-00499-0 (PMC12306013; doi:10.1186/s40035-025-00499-0)
Supplement: Supplementary file 1 — Additional file 1 (PDF 7263 KB) Table S1. Antibodies and primers. Fig. S1. hnRNP R regulates axonal tau levels. Fig. S2. hnRNP R is expressed in various regions of mouse brain. Fig. S3. Comparison of MAPT-ASO1 and -ASO2 treatment for reduction of axonal Mapt mRNA levels. Fig. S4. Comparison of MAPT-ASO1 and -ASO2 treatment for reduction of axonal tau levels. Fig. S5. Treatment with MAPT-ASO2 reduces axonal tau levels in motoneurons. Fig. S6. MAPT-ASO2 treatment has no effect on axonal hnRNP R level. Fig. S7. Spontaneous calcium activity of hippocampal neurons reveals no differences between control, scr-treated and MAPT-ASO2-treated cells. Fig. S8. Screening of additional ASOs for reducing axonal Mapt mRNA levels. Fig. S9. Validation of additional MAPT-ASOs for axonal tau reduction. Fig. S10. MAPT-ASO2-mediated reduction of axonal tau in iPSC-derived human motoneurons. Fig. S11. Body weight of aCSF- and ASO-injected 5×FAD mice. Fig. S12. Tubulin proteins are largely unaffected by MAPT-ASO2 treatment. [file 40035_2025_499_MOESM1_ESM.pdf]

Supplementary Materials

Axonal tau reduction ameliorates tau and amyloid pathology in a mouse model of Alzheimer's disease

Abdolhossein Zare, Saeede Salehi, Jakob M. Bader, Anna-Lena Wiessler, Manuela Prokesch, Vincent Albrecht, Carmen Villmann, Matthias Mann, Michael Briese* and Michael Sendtner*

*Corresponding authors. Michael Briese, Briese_M@ukw.de; Michael Sendtner, Sendtner_M@ukw.de

This PDF file includes:

Tables S1

Figs. S1 to S12

Antibodies		
Guinea pig monoclonal anti-Iba1	Synaptic Systems	Cat# 234 308<br>RRID: AB_2924932
Mouse monoclonal anti-Puromycin	Sigma-Aldrich	Cat# MABE343<br>RRID: AB_2566826
Mouse monoclonal anti-GAPDH	EMD Millipore	Cat# CB1001<br>RRID: AB_2107426
Mouse monoclonal anti-α-Tubulin	Sigma-Aldrich	Cat# T5168<br>RRID: AB_477579
Rabbit polyclonal anti-hnRNP R	Abcam	Cat# ab30930<br>RRID: AB_2295532
Rabbit polyclonal anti-Tau	Sigma-Aldrich	Cat# T6402<br>RRID: AB_261728
Mouse monoclonal anti-β-Amyloid (6E10)	BioLegend	Cat# 803001<br>PRID: AB_2564653
Mouse monoclonal anti-Phospho-Tau (AT8)	Thermo Scientific	Cat# MN1020<br>RRID: AB_223647
Mouse monoclonal anti-Phospho-Tau (AT180)	Thermo Scientific	Cat# MN1040<br>RRID: AB_223649
Recombinant human anti-β-Tubulin 3 Antibody	Miltenyi Biotec	Cat# 130-131-158<br>RRID: AB_2928186
Mouse monoclonal anti-Tau46	Cell Signaling Technology	Cat# 4019<br>RRID: AB_10695394
Mouse monoclonal anti-α-Tubulin	Sigma-Aldrich	Cat# T5168<br>RRID: AB_477579
Chicken Polyclonal anti-GFP	Abcam	Cat# ab13970<br>RRID: AB_300798
Rabbit IgG Control	PeproTech	Cat# 500-P00<br>RRID: AB_2722620
Donkey Anti-Rabbit IgG (HRP)	Jackson ImmunoResearch	Cat# 711-035-152<br>RRID: AB_10015282
Mouse Anti-Rabbit IgG Antibody (HRP)	Jackson ImmunoResearch	Cat# 211-032-171<br>RRID: AB_2339149
Donkey Anti-Chicken-Alexa Fluor® 488	Jackson ImmunoResearch	Cat# 703-545-155<br>RRID: AB_2340375
Donkey anti-Rabbit-Alexa Fluor 647	Thermo Fisher Scientific	Cat# A31573<br>RRID: AB_2536183
Donkey anti-Mouse-Cy3	Jackson ImmunoResearch	Cat# 715-165-150<br>RRID: AB_2340813
Donkey anti-Guinea Pig-Cy5	Jackson ImmunoResearch	Cat# 706-175-148<br>RRID: AB_2340462

Oligonucleotides
Gapdh-qPCR primer<br>Fwd: 5'-GCAAATTCAACGGCACA -3'
Gapdh-qPCR primer<br>Rev: 5'-CACCAGTAGACTCCACGAC-3'
Mapt-qPCR primer<br>Fwd: 5'- AGGCTGAGAGATGGATGGGT-3'
Mapt-qPCR primer<br>Rev: 5'-GAGCTAGCTAGGACTGCTGC-3'
Mapt-qPCR primer<br>Fwd: 5'-AGCCCTAAGACTCCTCCAGG-3'
Mapt-qPCR primer<br>Rev: 5'-GTGTTGGTAGGGATGGGGTG-3'
Actb-qPCR primer<br>Fwd: 5'-GATGACCCAGATCATGTTT-3'
Acb-qPCR primer<br>Rev: 5'-CGTGAGGGAGAGCATAG-3'

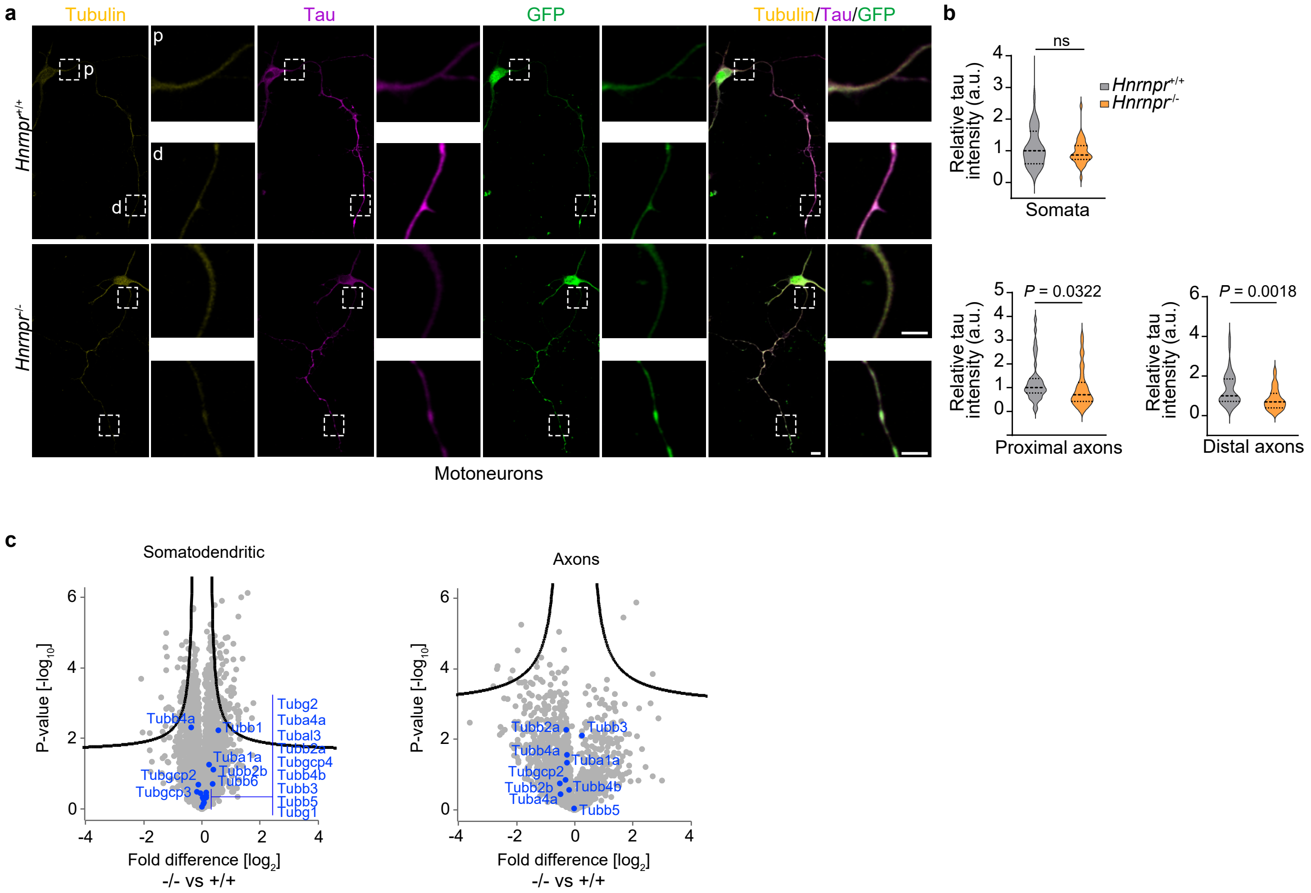

Fig. S1. hnRNP R regulates axonal tau levels. (**a**) Tau immunostaining of DIV 5 motoneurons cultured from *Hnrnpr*+/+ and *Hnrnpr*-/- mice, with proximal (p) and distal (d) regions of the axon marked. Motoneurons were transduced with an EGFP expression lentivirus for visualization of neuronal morphology and for normalization of tau levels. Scale bars: 10 µm and 5 µm (inset). (**b**) Tau immunointensities in the somata, and proximal and distal axonal regions of *Hnrnpr*+/+ and *Hnrnpr*-/- motoneurons. *n* = 35 to 37 motoneurons from three biological replicates. Mann-Whitney test. (**c**) Volcano plots showing protein alterations in axonal and somatodendritic compartments of *Hnrnpr*-/- compared to +/+ motoneurons cultured in microfluidic chambers from four biological replicates. Unpaired two-sided Student's t test. Tubulins are labelled in blue.

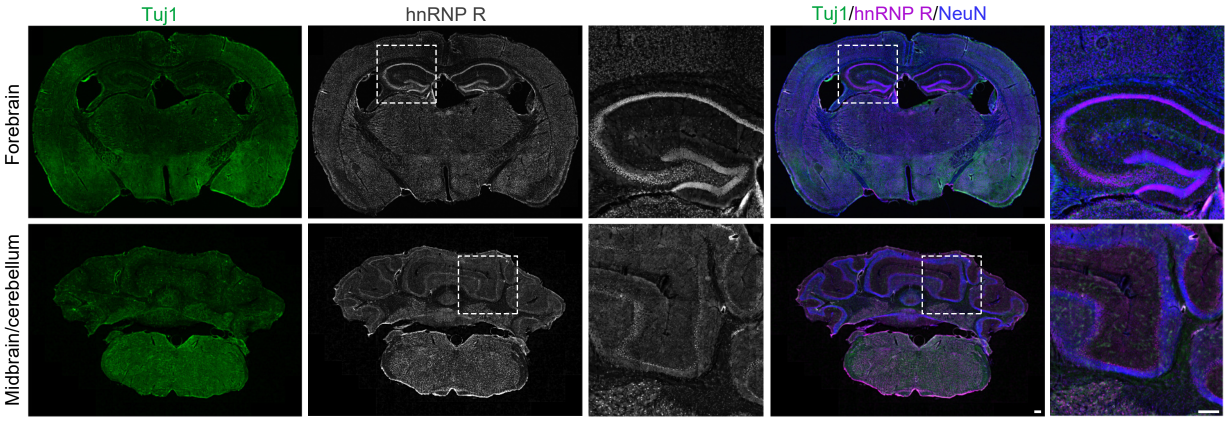

Fig. S2. hnRNP R is expressed in various regions of mouse brain. Immunohistochemical detection of hnRNP R in adult mouse brain. Scale bar: 200 µm.

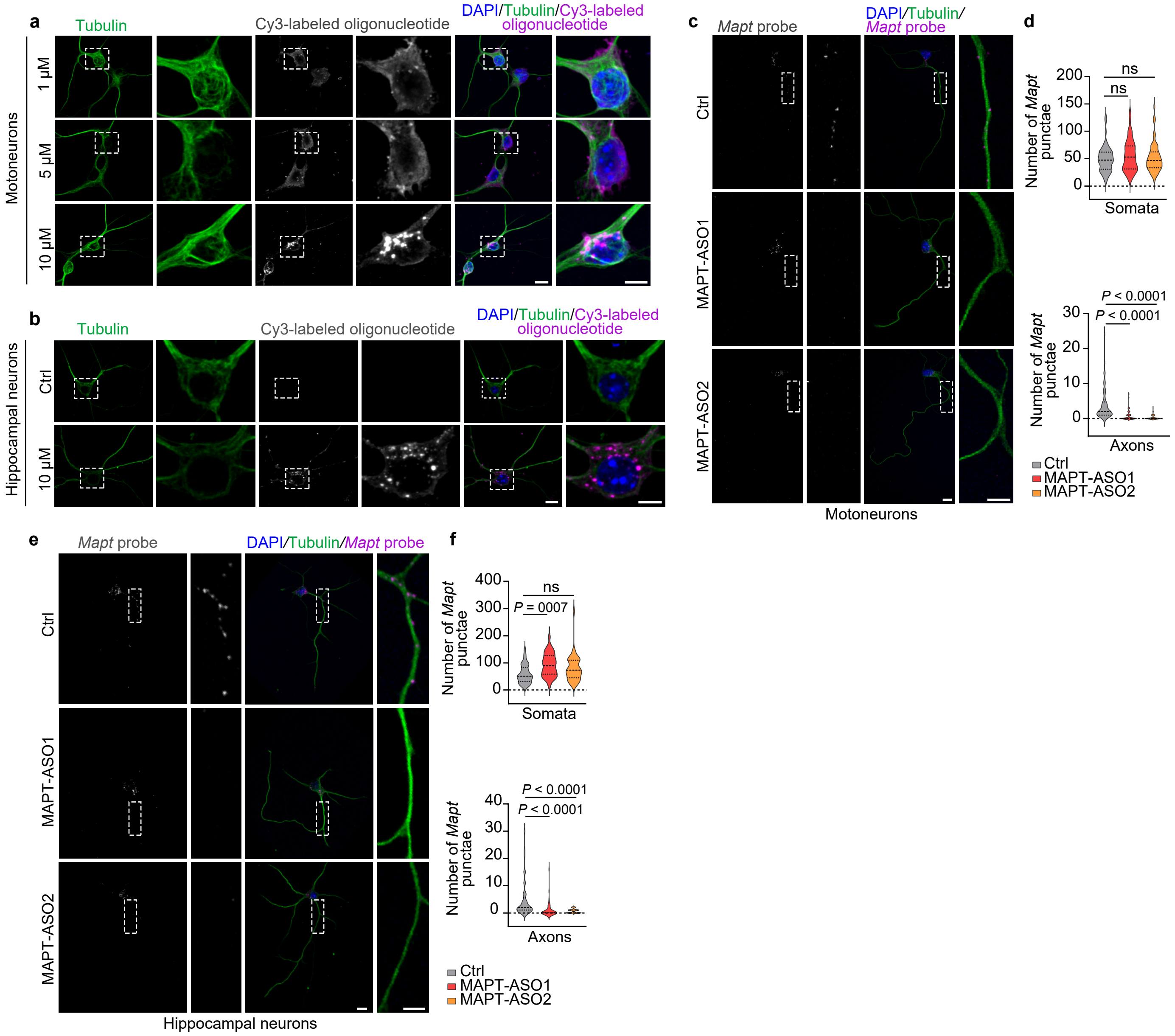

Fig. S3. Comparison of MAPT-ASO1 and -ASO2 treatment for reduction of axonal *Mapt* mRNA levels. (**a**) Immunofluorescence imaging of DIV 6 mouse motoneurons treated with different concentrations of a Cy3-labeled sense oligonucleotide. Scale bars: 10 μm and 5 μm (inset). (**b**) Immunofluorescence imaging of DIV 25 untreated (Ctrl) mouse hippocampal neurons and hippocampal neurons treated with 10 μM of a Cy3-labeled sense oligonucleotide. Scale bars: 10 μm and 5 μm (inset). Images in (**a**) and (**b**) are representative of at least three biological replicates. (**c**) *Mapt* FISH of DIV 6 untreated (Ctrl) mouse motoneurons and motoneurons treated with MAPT-ASO1 or -ASO2. Scale bars: 10 μm and 5 μm (inset). (**d**) Number of *Mapt* FISH punctae in the somata and axons of motoneurons. n = 46 to 56 motoneurons from three biological replicates. Kruskal Wallis with Dunn's multiple comparisons test. (**e**) *Mapt* FISH of DIV 6 untreated (Ctrl) mouse hippocampal neurons and hippocampal neurons treated with MAPT-ASO1 or -ASO2. Scale bars: 10 μm and 5 μm (inset). (**f**) Number of *Mapt* FISH punctae in the somata and axons of hippocampal neurons. n = 39 to 44 hippocampal neurons from three biological replicates. Kruskal Wallis with Dunn's multiple comparisons test.

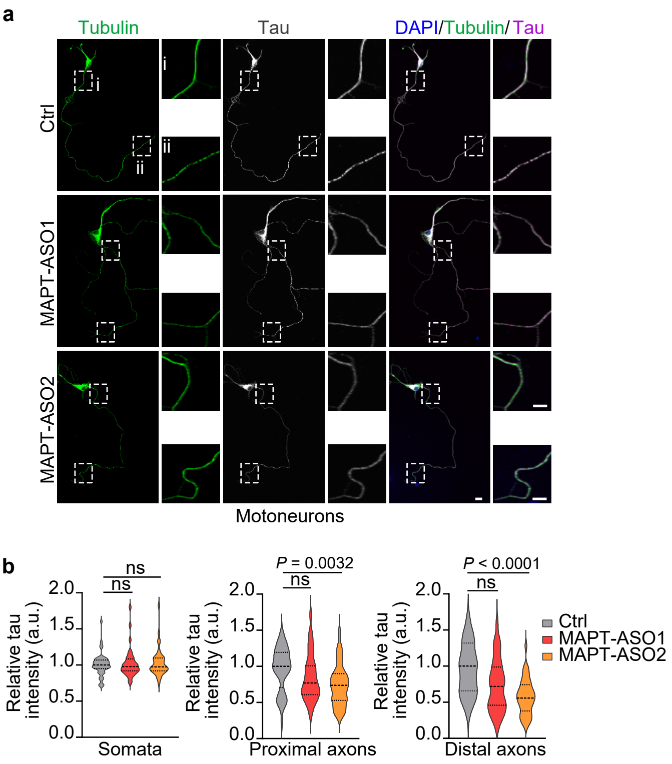

Fig. S4. Comparison of MAPT-ASO1 and -ASO2 treatment for reduction of axonal tau levels. (**a**) Total tau immunostain-ing of DIV 11 untreated (Ctrl) mouse motoneurons and motoneurons treated with MAPT-ASO1 or -ASO2, with proximal (i) and distal (ii) regions of the axon marked. Scale bars: 10 µm and 5 µm (inset). (**b**) Tau immunointensities in the somata and proximal and distal axonal regions of untreated and treated motoneurons. *n* = 35 to 46 motoneurons from three biological replicates. Kruskal Wallis with Dunn's multiple comparisons test. (**c**) Total tau immunostaining of DIV 22 untreated (Ctrl) mouse hippocampal neurons and hippocampal neurons treated with MAPT-ASO1 or -ASO2. Scale bars: 10 µm and 5 µm (inset). (**d**) Tau immunointensities in the somata and axons of untreated and treated hippocampal neurons. *n* = 35 to 46 hippocampal neurons from three biological replicates. Kruskal Wallis with Dunn's multiple comparisons test.

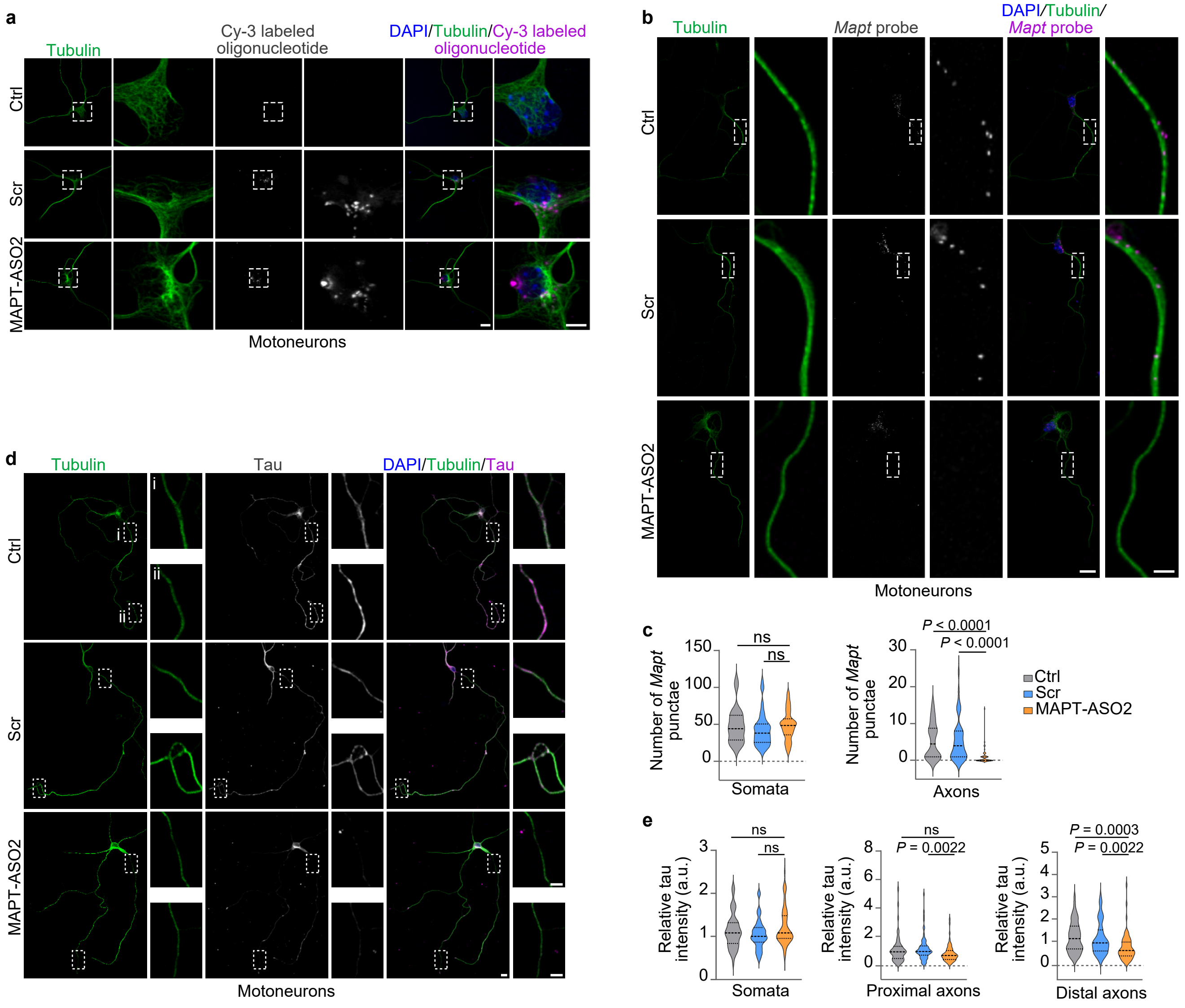

Fig. S5. Treatment with MAPT-ASO2 reduces axonal tau levels in motoneurons. (**a**) Immunofluorescence imaging of DIV 6 untreated (Ctrl) mouse motoneurons and motoneurons treated with 10 µM of a Cy3-labeled scramble oligonucle-otide as control or MAPT-ASO2. Scale bars: 10 µm and 5 µm (inset). Images are representative of at least three indepen-dent experiments. (**b**) *Mapt* FISH of DIV 6 untreated (Ctrl) mouse motoneurons and motoneurons treated with scramble oligonucleotide or MAPT-ASO2. Scale bars: 10 µm and 5 µm (inset). (**c**) Number of *Mapt* FISH punctae in the somata and axons of motoneurons. n = 36 to 46 motoneurons from three biological replicates. Kruskal Wallis with Dunn's multiple comparisons test. (**d**) Total tau immunostaining of DIV 11 untreated (Ctrl) mouse motoneurons and moto-neurons treated with scramble oligonucleotide or MAPT-ASO2, with proximal (i) and distal (ii) regions of the axon marked. Scale bars: 10 µm and 5 µm (inset). (**e**) Tau immunointensities in the somata and proximal and distal axonal regions of motoneurons. n = 54 to 65 motoneurons from five biological replicates. Kruskal Wallis with Dunn's multiple comparisons test.

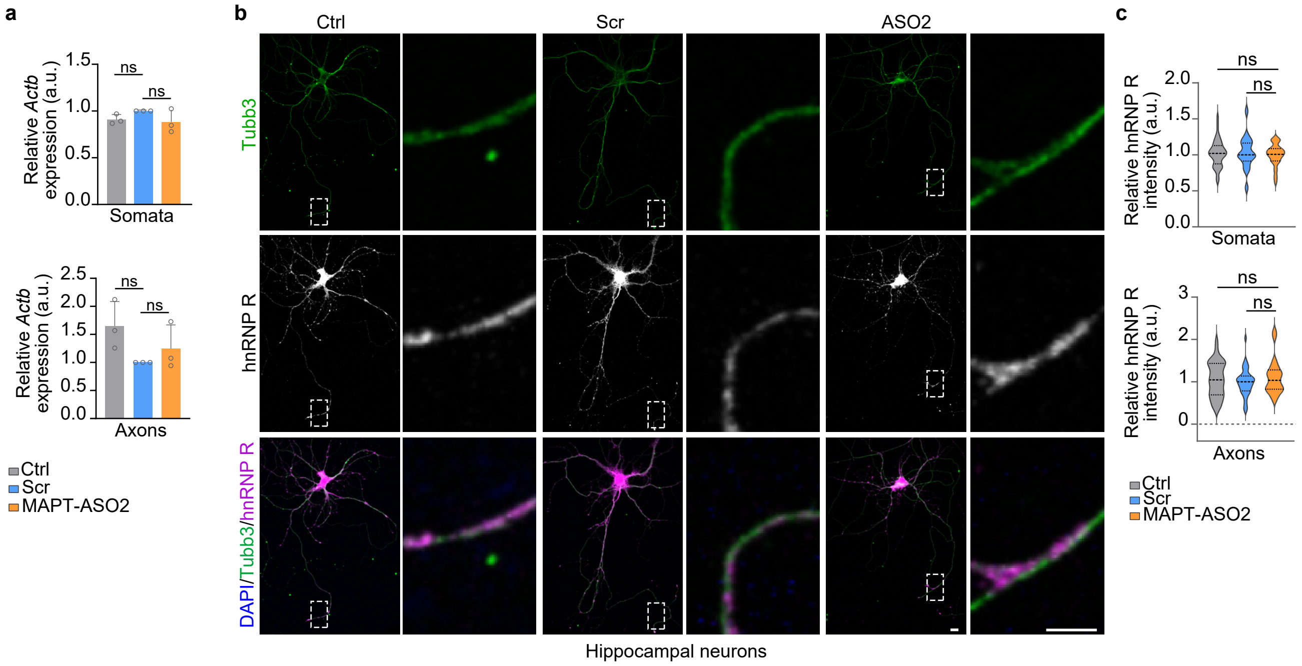

Fig. S6. MAPT-ASO2 treatment has no effect on axonal hnRNP R level. (**a**) qPCR analysis of *Actb* mRNA from somatodendritic and axonal RNA of compartmentalized DIV 7 cortical neurons treated with scramble oligonucleotide or MAPT-ASO2. Two-tailed one-sample t test. Data are mean ± s.d. of n = 3 biological replicates. (**b**) hnRNP R immunostaining of DIV 25 untreated (Ctrl) mouse hippocampal neurons and hippocampal neurons treated with scramble oligonucleotide or MAPT-ASO2, with distal regions of the axon marked. Scale bars: 10 µm and 5 µm (inset). (**c**) hnRNP R immunointensities in the somata and distal axonal regions of hippocampal neurons. n = 31 to 33 hippocampal neurons from three biological replicates. One-way ANOVA with Tukey's multiple comparisons test.

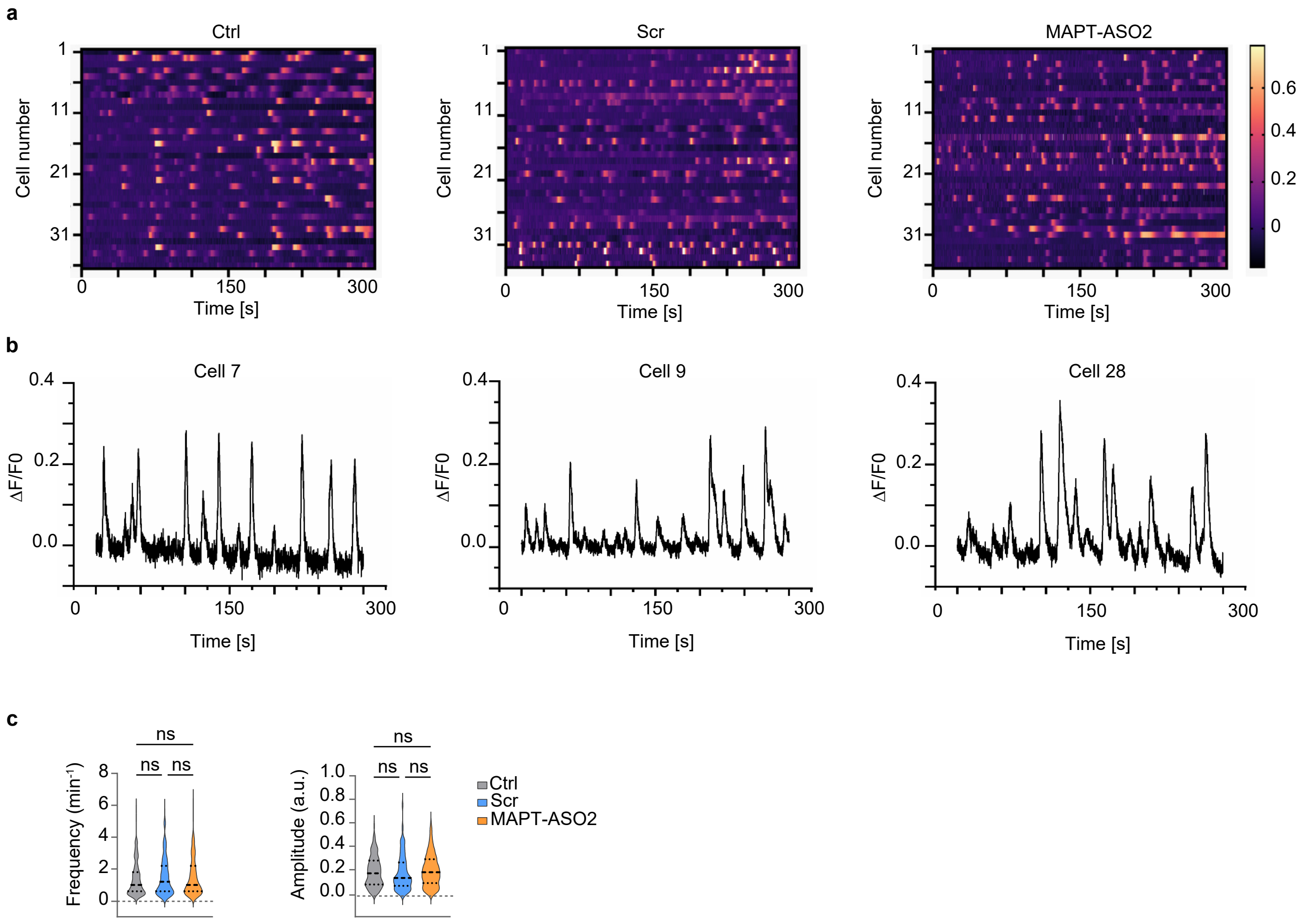

Fig. S7. Spontaneous calcium activity of hippocampal neurons reveals no differences between control, scr-treated and MAPT-ASO2 treated cells. (a) Neuronal activity from 34-36 cells of each condition is shown in a heatmap. (**b**) Activity graphs of exemplary ROIs for each condition. (**c**) Frequency (left) and amplitude (right) of spontaneous calcium activity events from n = 135-162 cells. Kruskal Wallis with Dunn's multiple comparisons test.

a

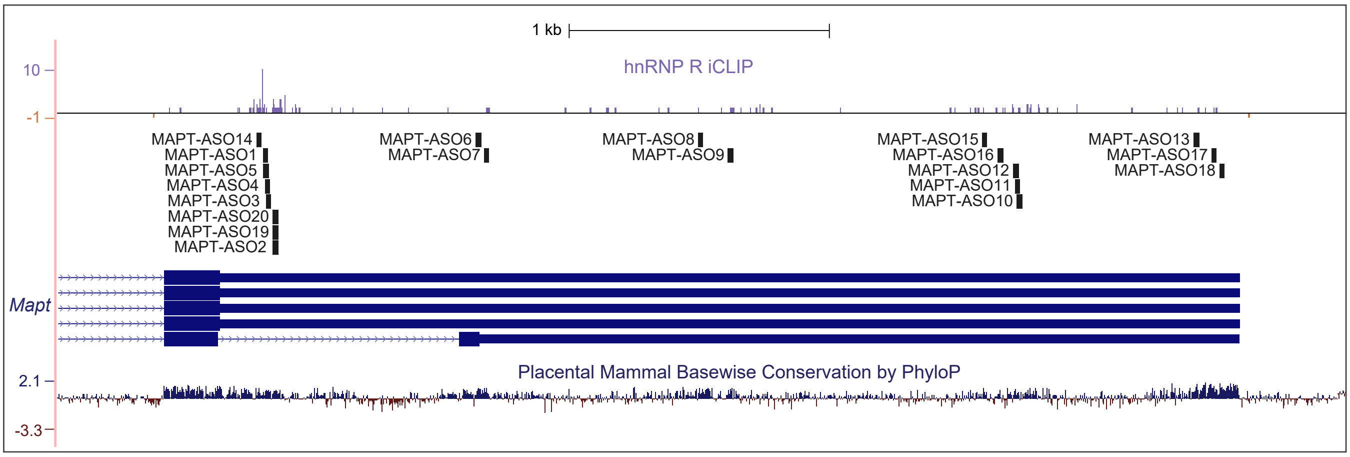

b

ASO name	Sequence	Start position in NG_007398.2	End position in NG_007398.2	Relative *Mapt* expression soma	Relative *Mapt* expression axon
MAPT-ASO1	TTTTGAAGTCCCGAGCCAAA	134864	134883	1.69	**0.40**
MAPT-ASO2	TCAATTTGGAAAGATGAAAT	134906	134925	1.38	**0.31**
MAPT-ASO3	TCCCATCACTGATTTTGAAG	134876	134895	1.20	**1.11**
MAPT-ASO4	ATCACTGATTTTGAAGTCCC	134872	134891	1.78	**0.70**
MAPT-ASO5	TGATTTTGAAGTCCCGAGCC	134867	134886	1.26	**0.17**
MAPT-ASO6	TTAGCCCTAAAGTCCCAGGT	135691	135710	1.17	**0.66**
MAPT-ASO7	CCAAGAGGCACAAGTCCTTA	135724	135743	0.83	**0.75**
MAPT-ASO8	CAGACAAATCCAACTACAAC	136566	136585	1.06	**0.50**
MAPT-ASO9	ATTTCAAGATACATGCGTCC	136645	136664	1.14	**0.94**
MAPT-ASO10	TGAAGTCAATTTAAATGGAA	138016	138035	1.23	**0.81**
MAPT-ASO11	TTTAAATGGAACTATTGATA	138007	138026	1.49	**0.32**
MAPT-ASO12	TGGAACTATTGATAAAGTGA	138001	138020	1.65	**0.51**
MAPT-ASO13	AAGAAATCATGGGACTTGCA	138696	138715	1.31	**0.72**
MAPT-ASO14	ACAAAAGCAGGTTA	134842	134855	0.77	**0.41**
MAPT-ASO15	GGGGGATTGTCCTCA	137878	137892	0.65	**0.38**
MAPT-ASO16	CTATCTAGCCCACCCAA	137949	137965	1.17	**0.99**
MAPT-ASO17	GACATTCACAGACAG	138768	138782	1.06	**0.47**
MAPT-ASO18	ATCATTTGTTAAAACACA	138797	138814	1.12	**0.45**
MAPT-ASO19	CAATTTGGAAAGATGAAA	134907	134924	0.98	**0.32**
MAPT-ASO20	AATTTGGAAAGATGAA	134908	134923	0.99	**0.23**

c

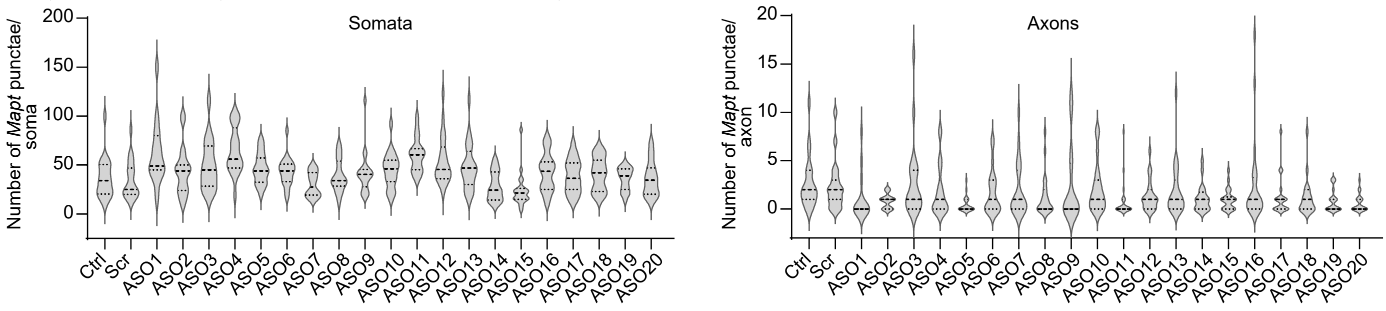

Fig. S8. Screening of additional ASOs for reducing axonal *Mapt* mRNA levels. (**a**) Binding sites of MAPT-ASO1 to -ASO20 in the *Mapt* 3' UTR. (**b**) Sequences of MAPT-ASO1 to -ASO20 and their binding positions along human *MAPT* NCBI Reference Sequence NG_007398.2. *Mapt* mRNA levels were quantified by FISH in somata and axons of ASO-treated mouse hippocampal neurons and normalized to untreated hippocampal neurons. MAPT-ASOs with >50% reduction of axonal *Mapt* mRNA levels are highlighted in yellow. (**c**) Number of *Mapt* FISH punctae in the somata and axons of hippocampal neurons at DIV 6. *n* = 15 to 21 hippocampal neurons. Kruskal Wallis with Dunn's multiple comparisons test.

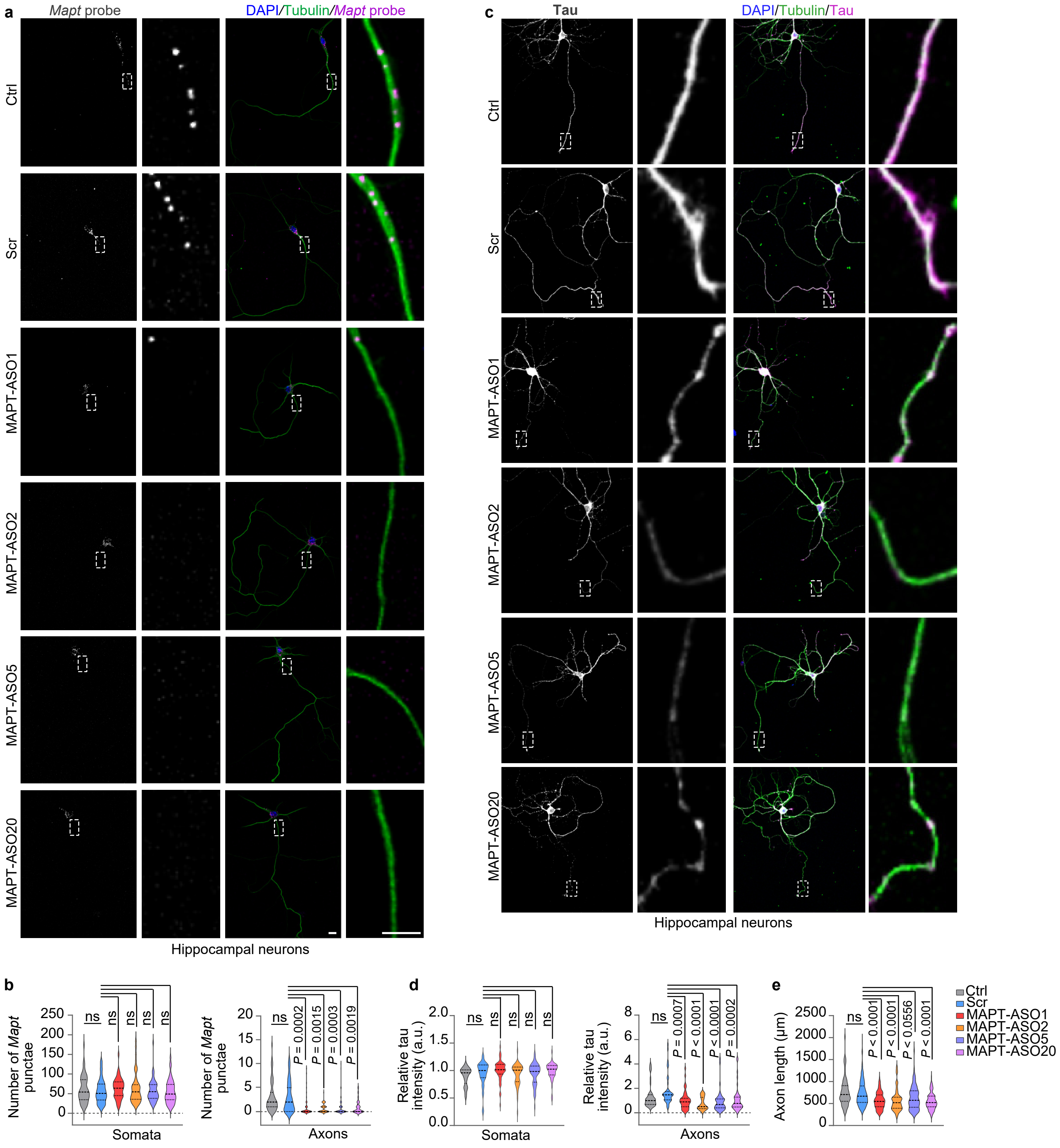

Fig. S9. Validation of additional MAPT-ASOs for axonal tau reduction. (**a**) *Mapt* FISH of DIV 6 untreated (Ctrl) mouse hippocampal neurons and hippocampal neurons treated with scramble oligonucleotide or MAPT-ASO1, 2, 5 or 20. Scale bars: 10 µm and 5 µm (inset). (**b**) Number of *Mapt* FISH punctae in the somata and axons of hippocampal neurons. n = 44 to 57 hippocampal neurons from three biological replicates. Kruskal Wallis with Dunn's multiple comparisons test. (**c**) Total tau immunostaining of DIV 25 untreated (Ctrl) mouse hippocampal neurons and hippocampal neurons treated with scramble oligonucleotide or MAPT-ASO1,2,5 or 20 with distal regions of the axon marked. Scale bars: 10 µm and 5 µm (inset). (**d**) Total tau immunointensities in the somata and distal axonal regions of DIV 25 hippocampal neurons. n = 30 to 46 hippocampal neurons from three biological replicates. Kruskal Wallis with Dunn's multiple comparisons test. (**e**) Axon lengths of DIV 25 hippocampal neurons. n = 81 to 179 hippocampal neurons from three biological replicates. Kruskal Wallis with Dunn's multiple comparisons test.

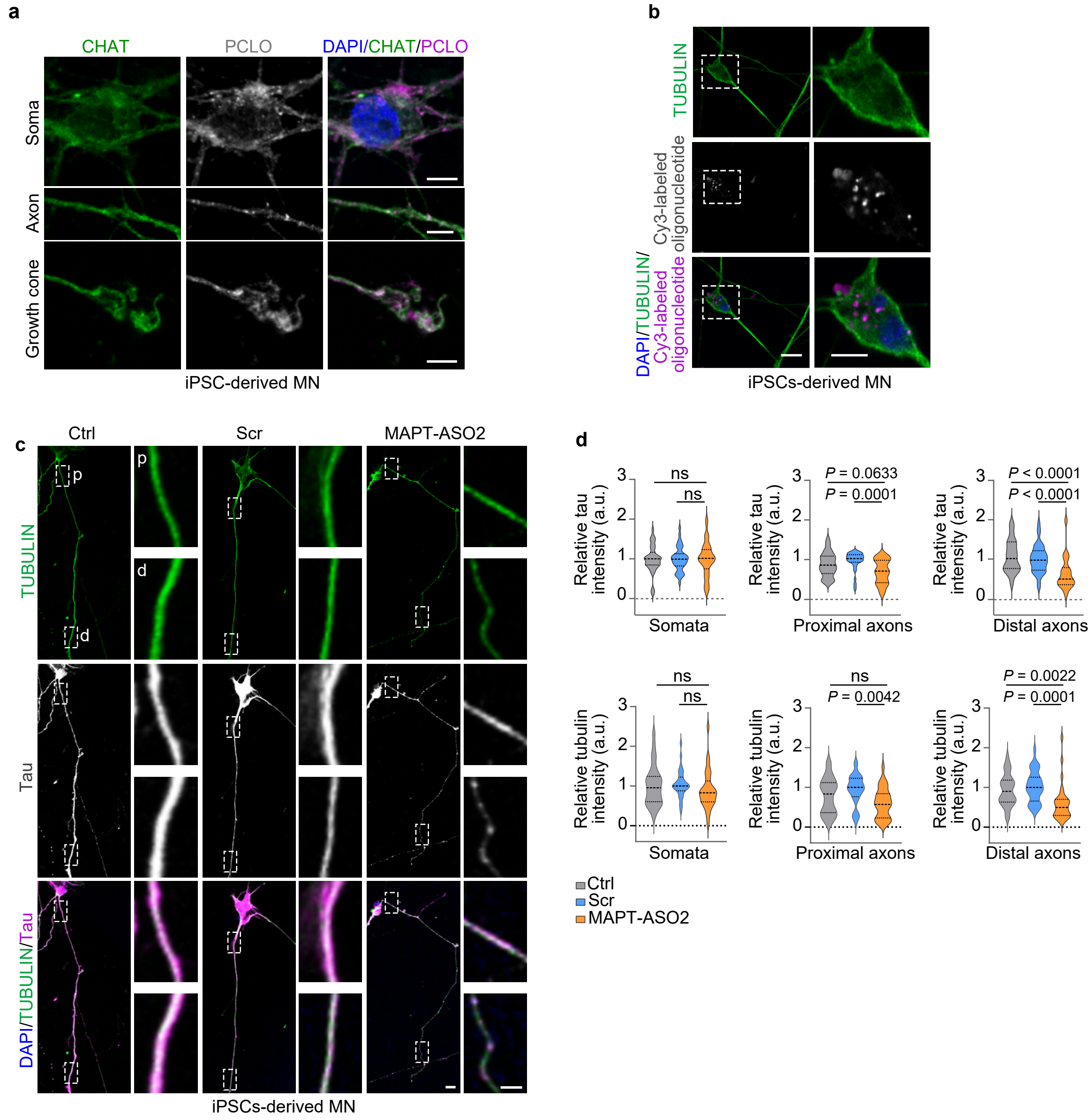

Fig. S10. MAPT-ASO2-mediated reduction of axonal tau in iPSC-derived human motoneurons. (**a**) Immunostaining of iPSC-derived human motoneurons (MN) cultured for 25 DIV for PICCOLO (PCLO) and choline acetyltransferase (CHAT). Scale bars: 5 µm. (**b**) Immunofluorescence imaging of iPSC-derived human motoneurons treated with 10 µM of a Cy3-labeled scramble oligonucleotide and cultured for 21 DIV. Scale bars: 10 µm and 5 µm (inset). (**c**) Total tau immunostaining of untreated (Ctrl) iPSC-derived human motoneurons and iPSC-derived human motoneurons treated with scramble oligonucleotide or MAPT-ASO2, with proximal (p) and distal (d) regions of the axon marked. Scale bars: 10 µm and 5 µm (inset). (**d**) Total tau immunointensities in the somata and proximal and distal axonal regions of iPSC-derived human motoneurons. n = 38 to 39 iPSC-derived human motoneurons from four biological replicates. Kruskal Wallis with Dunn's multiple comparisons test.

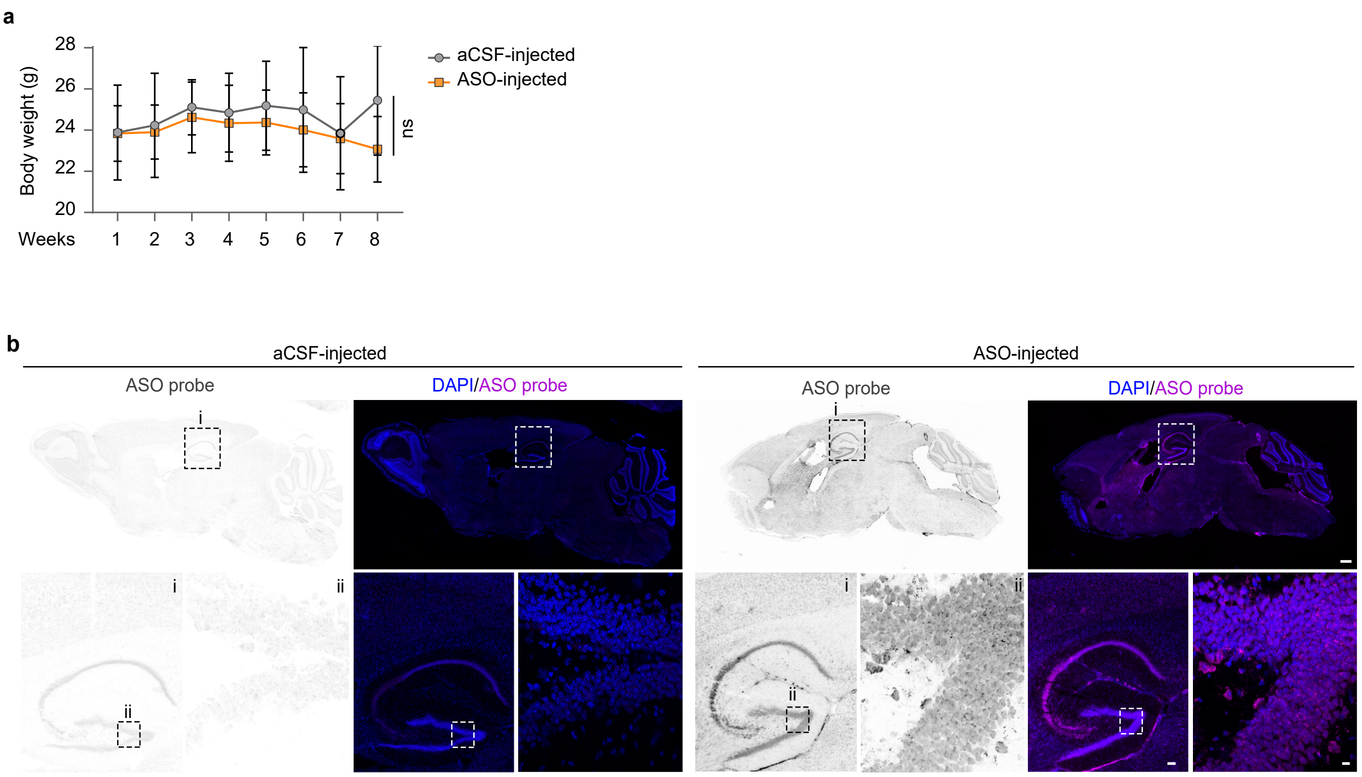

Fig. S11. Body weight of aCSF- and ASO-injected 5XFAD mice. (**a**) Body weight of aCSF- and ASO-injected 5XFAD mice at different time points. *n* = 12 to 14 mice. Two-way repeated measures ANOVA with Sidak's multiple comparisons test. (**b**) FISH for MAPT-ASO2 detection in sagittal brain sections of 5xFAD mice treated with aCSF or MAPT-ASO2. Scale bars: 500 µm, 100 µm (inset i) and 10 µm (inset ii).

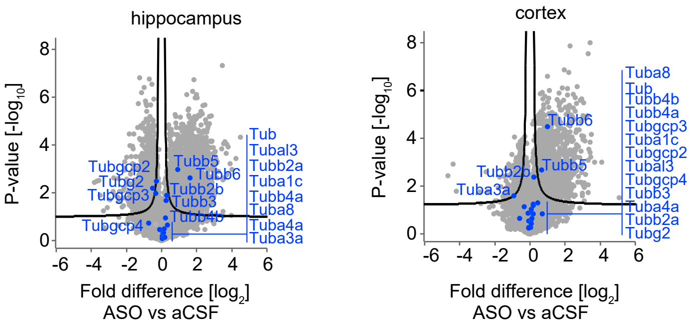

Fig. S12. Tubulin proteins are largely unaffected by MAPT-ASO2 treatment. Volcano plots showing protein alterations in in hippocampus and cortex of aCSF- and ASO-injected 5XFAD. Unpaired two-sided Student's t test. Tubulins are labelled in blue.
