# Supplementary figures and images for: Axonal tau reduction ameliorates tau and amyloid pathology in a mouse model of Alzheimer’s disease
Source: Transl Neurodegener. 2025 Jul 29;14:39. doi: 10.1186/s40035-025-00499-0 (PMC12306013; doi:10.1186/s40035-025-00499-0)

## UNCROPPED IMMUNOBLOTS

Fig. 4j

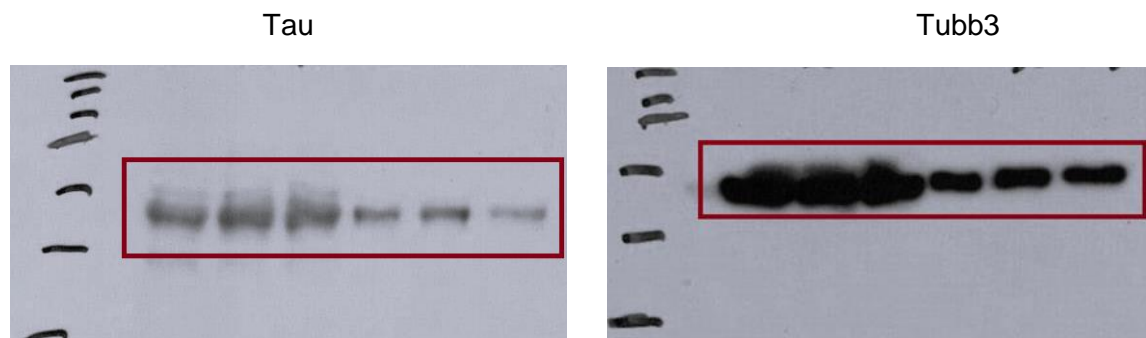

Fig. 6g

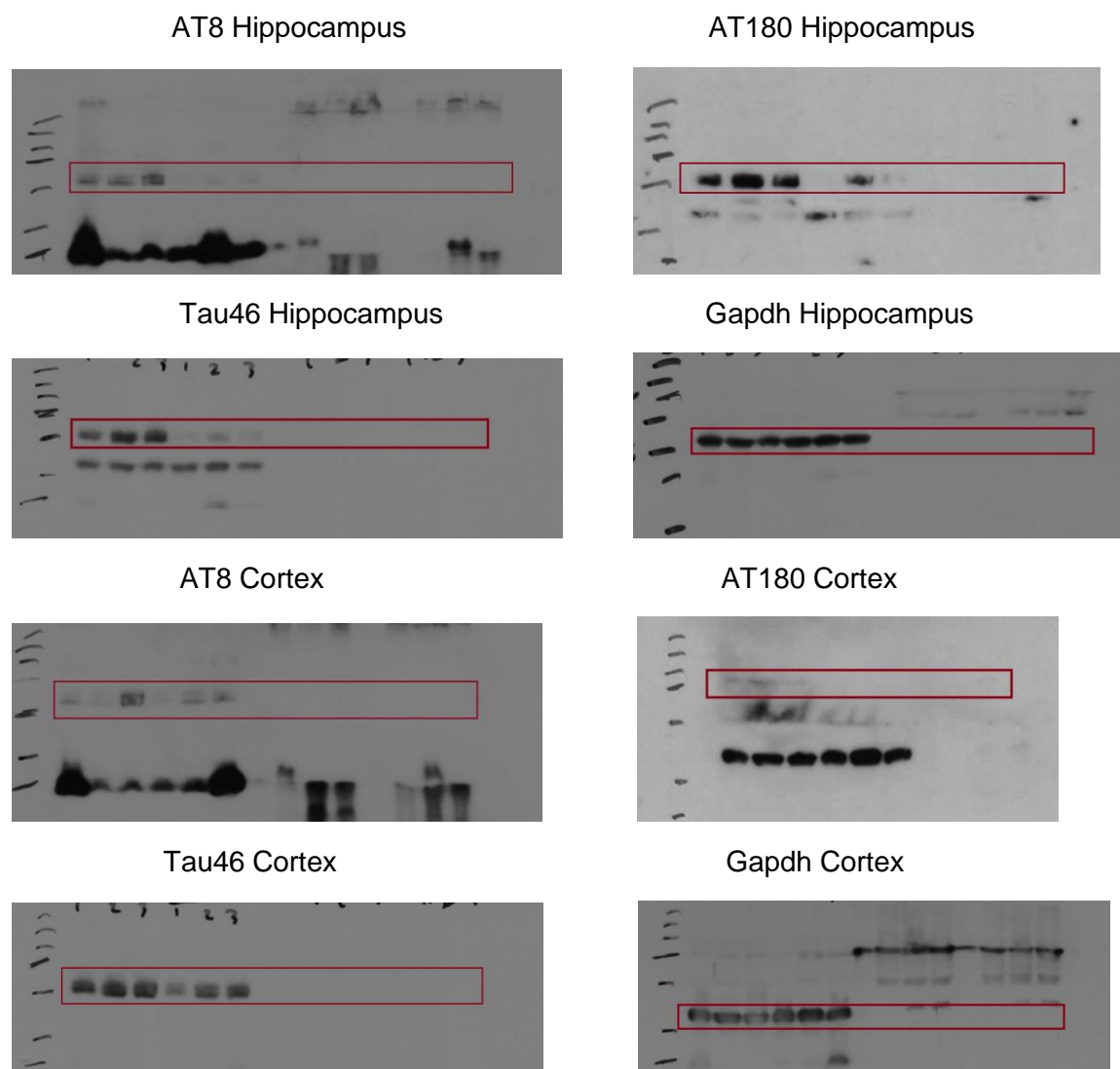

Supplement: Supplementary file 3 — Additional file 3 (PDF 91 KB) Uncropped immunoblot images. [file 40035_2025_499_MOESM3_ESM.pdf]
